# Developing a one-shot medical Spanish module for medical students: an open educational resource approach

**DOI:** 10.3389/fmed.2026.1957506

**Published:** 2026-09-17

**Authors:** Juli McCarroll

**Affiliations:** Department of Medical Library, Western Michigan University Homer Stryker M.D. School of Medicine, Kalamazoo, MI, United States

**Keywords:** curriculum, health equity, language concordance, medical education, medical school, medical Spanish, medical students, open educational resources

## Abstract

One-shot instruction sessions, commonly used in academic libraries, are introductory presentations that teach research skills, source evaluation, and database use. They are designed to make a meaningful impact in a very limited time and are motivational rather than comprehensive. Similarly, one-time medical Spanish workshops in medical education can serve as initial learning experiences for medical students that encourage continued engagement with language learning to build fluency and ultimately improve health equity for Spanish-speaking patients. This paper presents the development and delivery of a one-hour, open educational resource medical Spanish module that can be distributed and adapted for personal use. A medical librarian developed and delivered a one-hour medical Spanish module to first-year medical students (*N* = 84). Learning was assessed using two questions aligned with the session's objectives that were embedded in a summative course examination. Descriptive analysis was used to summarize student responses and evaluate understanding of key concepts. On the summative examination administered one month later, approximately three-fifths of students correctly answered the module-related questions. Additionally, about 39% of the class pursued further Spanish learning opportunities during medical school. This educational intervention suggests that brief, introductory medical Spanish instruction may support early development of cultural and linguistic competencies and encourage continued study. While limited in scope, such exposure may serve as a gateway to more sustained engagement with the language.

## Introduction

Effective communication with patients who have limited English proficiency is essential to high-quality healthcare. Spanish is the most commonly spoken non-English language in the United States (1, 2) yet a shortage of Spanish-speaking healthcare providers contributes to language discordance between patients and clinicians, which has been associated with increased misdiagnoses, adverse events, miscommunication, lower levels of trust, and worse healthcare outcomes (3–10). Spanish language and culture educational interventions during medical training can lead to an increased number of providers who are better prepared to engage in language-concordant care within their scope of competence to provide optimal health outcomes and advance health equity (11–34).

Although numerous medium and longitudinal-duration medical Spanish programs have been described (35), few reports have examined the educational value of single-session interventions. Nevertheless, evidence suggests that even brief medical Spanish interventions can produce meaningful educational benefits (16, 17, 19, 22, 25, 28, 30, 31, 36, 37). A useful framework for conceptualizing brief medical Spanish interventions comes from academic librarianship, where one-shot instruction sessions are widely used to introduce learners to foundational concepts such as research skills, source evaluation, and database use. Although not intended to provide comprehensive mastery, one-shot instruction sessions can increase awareness, motivate continued learning, and foster ongoing engagement with educational resources and librarians (38–42). Similarly, one-shot medical Spanish workshops in medical education can provide targeted language exposure and increase learner interest in continued language and cultural education to support the development of skills associated with more effective communication and language-concordant care. Educational approaches that increase students’ knowledge of the languages and cultures of Spanish-speaking patients and promote language concordance warrant further consideration. Open educational resources may facilitate broader implementation by reducing cost, preparation time, and barriers to curricular adoption since they can be freely accessed and adapted (43–45). Such approaches may be particularly valuable for institutions that lack the resources required to develop longitudinal medical Spanish curricula.

Despite growing interest in medical Spanish education, our review of the published literature identified only one report describing a short, single-session medical Spanish intervention in medical school, suggesting that this educational approach remains underrepresented (46). This paper describes the development, implementation, and preliminary educational outcomes of a one-hour OER medical Spanish workshop delivered at Western Michigan University Homer Stryker M.D. School of Medicine (WMed). Designed as a scalable, low-resource intervention, the workshop introduces foundational medical Spanish concepts while encouraging continued language learning and cultural knowledge among medical students.

## Pedagogical framework

A time-sensitive opportunity arose when WMed leadership identified an available curricular slot for first-year medical students, allowing for the development and delivery of a one-hour educational session. There was interest in and demand for language-focused programming instruction because a previously required medical Spanish course had been phased out during curricular revisions.

To address this need, a librarian with Spanish-language proficiency and academic training in teaching, languages, and linguistics was invited to develop and deliver an introductory medical Spanish session. The goal of the module was not to achieve language proficiency, but rather to introduce foundational medical Spanish concepts and encourage students to pursue additional language-learning opportunities available through the medical school and local community. Learning was assessed through two questions on the subsequent summative examination, while continued engagement was assessed by tracking the number of students who subsequently enrolled in another Spanish elective at the school. Leadership provided several resources to include in the presentation, while the librarian designed and developed the remainder of the instructional content.

The module content was informed by a review of the medical Spanish and language-concordant care literature. Given the limited instructional time available, content was selected to provide an introduction to foundational concepts that support future language learning and culturally responsive patient care. Five major topics were identified for inclusion: (1) the importance of medical Spanish in healthcare, (2) patient safety and language proficiency limitations, (3) cultural humility, (4) practical communication skills, and (5) resources for continued learning.

The importance of medical Spanish was emphasized through discussion of the prevalence of Spanish-speaking patients in the United States and the relationship between language concordance and patient communication, trust, satisfaction, adherence, and health outcomes. Patient safety content focused on recognizing personal language limitations and understanding the appropriate roles of professional medical interpreters. Students were encouraged to view medical Spanish as a skill that can enhance communication while recognizing situations in which interpreter services are necessary. Cultural humility was introduced as a framework for engaging patients with respect and openness while acknowledging the limitations of one's own knowledge and experiences. Students were encouraged to recognize that language concordance alone does not eliminate cultural differences or guarantee shared understanding. Practical communication skills were prioritized over vocabulary memorization and focused on common clinical interactions, including greetings, introductions, obtaining consent, assessing pain, discussing medications, taking a history, providing education, and confirming understanding. The module also emphasized that medical Spanish extends beyond direct translation and requires cultural humility, including respectful communication, avoidance of assumptions based on language or culture, and awareness of sociocultural influences on health. Not all topics could be covered in the timeframe; therefore, those included were selected to introduce core principles that students could build upon through additional coursework, self-directed learning, and clinical experience. Because the session was designed as an introductory intervention rather than a comprehensive language course, students were provided with resources to support continued learning. These included language-learning applications and medical Spanish-specific educational tools including CanopySpeak, Google Translate, and SpanishDict, elective coursework, volunteering opportunities, and community engagement activities.

The instructional design was guided by the American Council on the Teaching of Foreign Languages (ACTFL) World-Readiness Standards (47) and relevant Liaison Committee on Medical Education (LCME) standards related to communication skills, cultural competence, structural competence, and health equity (48). The ACTFL framework emphasizes communication, cultural understanding, and continued language development, while LCME standards support the integration of patient-centered communication and culturally responsive care concepts.

Language-learning activities incorporated principles from established educational frameworks, including Gradual Release of Responsibility (GRR) (49–51), Presentation-Practice-Production (PPP) (52, 53), scaffolding (54–57), and choral repetition and drilling (58). These approaches were selected because they emphasize instructor modeling, guided practice, active learner participation, and progressive independence in language use. To support engagement within the limited instructional time available, the module also incorporated active learning strategies and a culturally responsive game-based learning activity to reinforce medical Spanish concepts.

Drawing from principles commonly used in academic library one-shot instruction, the module incorporated active learning strategies designed for brief educational encounters, including focused learning objectives, learner engagement, and opportunities for participation and feedback (40–44). Because the session was limited to one hour, instructional activities were intentionally designed to maximize interaction rather than rely solely on didactic presentation.

## Learning environment

A culturally responsive game-based learning activity using a modified version of Mexican lotería was incorporated to reinforce medical Spanish concepts and provide students with an interactive opportunity to practice vocabulary in a low-stakes environment. A lotería-inspired game was selected because it is a culturally familiar game in many Spanish-speaking communities and provided an engaging framework for introducing language concepts while exposing students to a culturally relevant educational tool. Gamification elements, including competition and achievement-based rewards, were incorporated to increase participation, encourage motivation, and promote engagement with the material (59–66). During the activity, students matched Spanish medical terms with corresponding images, reinforcing vocabulary through repetition, peer interaction, writing, and contextual learning while receiving immediate feedback from peers and instructors.

The lotería boards and other educational materials developed for this project are available under a Creative Commons Attribution–NonCommercial 4.0 International License (CC BY-NC 4.0), permitting noncommercial sharing, adaptation, and reuse with appropriate attribution and the license is available at https://creativecommons.org/licenses/by-nc/4.0/. The module was released under a Creative Commons license to facilitate adoption, adaptation, and dissemination by other health professions educators.

Guided by these educational standards and instructional frameworks, a one-hour, in-person medical Spanish module was delivered to first-year medical students (*N* = 84). The session combined brief didactic instruction with active learning strategies designed to introduce foundational medical Spanish concepts, promote learner engagement, and encourage continued language development. Didactic content included an overview of the role of Spanish in U.S. healthcare, common health concerns affecting Spanish-speaking patients, the importance of medical Spanish in patient care, resources for translation and language support, and recommendations for continued learning.

To reinforce key concepts, students participated in several solo and group-based interactive learning activities that were intentionally chosen because these approaches have been shown to improve engagement and retention during brief educational interventions. A lotería-style game-based activity was used to strengthen recognition and recall of basic anatomical terminology in Spanish. Students also engaged in a guided patient–physician dialogue that modeled effective clinical communication strategies and highlighted appropriate use of language skills in patient encounters. Finally, a matching exercise allowed students to apply concepts related to cultural considerations while providing an opportunity for immediate feedback and discussion. These activities were selected to encourage active participation and practical application of the material within the constraints of a one-hour instructional session.

All instructional materials are available in the Supplementary Materials, including the PowerPoint presentation (Supplementary File 1) and the lotería boards with links to the public domain images used from Wikimedia Commons, patient-physician dialogue, matching exercise, and speaker notes (Supplementary File 2).

The module was designed to facilitate adaptation, reuse, and dissemination. The materials include a PowerPoint presentation and script, three lotería boards, a patient–physician dialogue, and a matching exercise. Educators may add, delete, or modify slides to align with local curricular objectives, learner needs, available instructional time, and Spanish language proficiency levels. All images used in the module were in the public domain, eliminating licensing barriers to reuse. Detailed presenter notes are in Supplementary File 2 including the implementation instructions denoted by parentheses, reducing preparation time and supporting consistent delivery across instructors. The module was originally designed as a one-hour session but could be shortened or expanded by modifying the amount of guided practice, discussion, or cultural content included. No prior Spanish language knowledge is required of the students, making the module suitable for novice learners. The lotería activity includes three board variations and can be adapted to different healthcare topics, learner populations, or languages. Materials may be used in undergraduate medical education, graduate medical education, or other health professions programs and can be delivered in face-to-face, hybrid, or online learning environments. However, face-to-face delivery may provide advantages for choral repetition, pronunciation practice, peer interaction, immediate feedback, and guided communication exercises. The open educational design supports ongoing revision, localization, and customization by educators while maintaining the module's core learning objectives.

## Results and assessment

This activity did not constitute human subjects research requiring IRB review. The educational module was originally developed and delivered as a presentation. Its current use is limited to a case study for educational purposes, not research. Therefore, IRB approval was not required.

Learning was assessed using two librarian–developed multiple-choice questions aligned with the module objectives and embedded within a summative course examination administered approximately one month after the session. Question 1 mapped to cultural understanding and recognition of a culturally specific health concept, while Question 2 mapped to basic clinical communication skills and appropriate patient greetings. Student performance on these items was analyzed descriptively to provide an indication of students’ ability to recognize selected medical Spanish concepts introduced during the session, not Spanish proficiency or clinical communication competency. Sixty-one percent of students answered Question 1 correctly, whereas 62% answered Question 2 correctly.

While it is possible that prior knowledge may explain performance on both items, individual-level data was not available, so performance stratified by prior proficiency could not be completed.

Although the one-shot module cannot be considered the cause of subsequent enrollment in medical Spanish electives, the continued engagement observed among some students is noteworthy. These findings suggest that introductory curricular experiences may provide an entry point for learners who go on to pursue additional medical Spanish education. Because students’ prior interest in Spanish and experiences with Spanish-speaking communities were not measured, however, it cannot be determined whether the module influenced these subsequent choices.

The questions included the following:

Question 1: Mr. García, a 40-year-old Latino patient, gets a 30-minute lunch break. Whether he is hungry or not, he typically drives to a fast-food restaurant and eats a burger, fries, and soda on his way back to work. His main complaints are stomachache, cramps, bloating, and diarrhea. Which of the following is the most likely cause?

- la diabetes
- los nervios
- la cuarentena
- el empacho

Answer: D

Question 2: You are meeting a new Latina patient who does not speak English, Ms. González. You want to greet her, introduce yourself, and ask her how she is feeling, in Spanish. What is the most appropriate option?

- Buenos días, señorita González. Soy el doctor Smith. ¿Cómo se siente?
- Buenos días, señora González. Soy el doctor Smith. ¿Cómo se siente?
- Buenos días, señorita González. Soy el doctor Smith. ¿Siente algún dolor?
- Buenos días, señora González. Soy el doctor Smith. ¿Siente algún dolor?

Answer: A

In Question 1, the use of “the most likely cause of the symptoms” is a diagnostic question that would have a different biomedical answer. For individuals reusing this module, more appropriate wording might be “Which culturally specific folk illness is this patient most likely to use to describe his symptoms?”

In Question 2, traditionally, *señorita* has been used for unmarried or younger women, whereas *señora* has been associated with married or older women; however, usage varies across Spanish-speaking regions and increasingly reflects age and context rather than marital status alone (67–69).

Records of the language background of this class indicate that there was American Medical College Application Service (AMCAS) language-specific data available for 71 students, of whom 31 students listed some level of Spanish language proficiency: 14 Basic, 8 Fair, 6 Good, 2 Advanced, and 1 Native/Functionally Native. The distribution of reported proficiency levels is available in Table 1. Additionally, there were non-language-specific data for six additional members of that class not represented above, including one Fluent in English Only, four Fluent in One Additional Language, and one fluent in two or More Additional Languages. This non-specific data is available in Table 2. Nearly all students were neither native nor advanced Spanish speakers.

**Table 1:** AMCAS language-specific data: Spanish proficiency.

Spanish proficiency level	Number of students	Percent of 31 students
Basic	14	45.2%
Fair	8	25.8%
Good	6	19.4%
Advanced	2	6.5%
Native/Functionally Native	1	3.2%
Total	31	100.0%

**Table 2:** Non-language-specific language background data.

Language background category	Number of students	Percent of 6 students
Fluent in English Only	1	16.7%
Fluent in One Additional Language	4	66.7%
Fluent in Two or More Additional Languages	1	16.7%
Total	6	100.0%

In addition, data on Spanish electives administered by the Medical Library that students registered for after the one-shot medical Spanish module were collected. Approximately 39% of the class enrolled in and completed a Beginning, Intermediate, or Advanced Medical Spanish with Canopy elective, and 2% took the WikiProject Medical Translation elective.

## Discussion

Medical schools often face competing curricular priorities and limited instructional time, creating challenges for integrating additional language and cultural competency training into already dense curricula. Brief, focused interventions may provide a feasible approach for introducing foundational medical Spanish concepts while creating pathways for learners to pursue longitudinal language development. This educational intervention suggests that a brief medical Spanish learning experience may contribute to the early development of linguistic awareness and culturally responsive communication skills while encouraging students to pursue additional language learning opportunities. Although a single one-hour session cannot establish clinical proficiency, early exposure may serve as an entry point by increasing awareness of the importance of language-concordant care and motivating students to seek further opportunities for skill development.

To explore subsequent engagement with medical Spanish education, enrollment data for Spanish electives offered by the Medical Library following completion of the module were reviewed, and a substantial proportion of students subsequently engaged in additional medical Spanish electives. Although participation in these electives cannot be directly attributed to the one-shot module, continued engagement with medical Spanish-related learning opportunities among a subset of learners suggests that brief introductory experiences may serve as an initial exposure point within a longitudinal pathway for medical Spanish education. It is also possible that students who pursued additional electives had a pre-existing interest in Spanish language learning or service with Spanish-speaking communities, which was not measured in this study.

This intervention was implemented in an institutional environment that provides multiple opportunities for learners to apply and expand their linguistic and cultural skills through curricular, extracurricular, and community-based experiences. These opportunities include international health electives in Peru, participation in the Latino Medical Student Association, activities recognizing National Hispanic Heritage Month, volunteering at clinics serving migrant workers, and student-led initiatives that support Spanish-speaking patients. Together, these opportunities highlight the importance of embedding brief educational interventions within a broader institutional framework that supports the longitudinal development of language skills, cultural humility, and equitable patient care.

The open educational design of this module may further support dissemination and adaptation by reducing barriers to implementation for institutions seeking to introduce medical Spanish education but lacking dedicated faculty time, curricular space, or financial resources. The ability to modify and reuse openly available materials allows educators to tailor instruction to local learner needs, incorporate institution-specific resources, and build on foundational concepts through more advanced language training. Brief interventions such as this may therefore serve as a scalable strategy for initiating medical Spanish education and supporting the development of future clinicians who are prepared to provide more linguistically responsive care.

## Constraints

Several limitations should be considered when interpreting these findings. First, although the OER format facilitates free access, adaptability, and dissemination, a single one-hour session cannot develop clinical Spanish proficiency or replace longitudinal language training. The module was designed to introduce foundational concepts related to communication with Spanish-speaking patients, cultural humility, patient safety, appropriate use of interpreter services, and available resources for continued learning. It was not intended to prepare students to provide language-concordant care independently. Medical Spanish proficiency requires repeated exposure, deliberate practice, and opportunities for authentic communication over time; therefore, a brief intervention is unlikely to produce sustained proficiency or long-term behavioral change independently. In addition, although language training can increase students’ confidence, increased confidence may not always reflect corresponding gains in proficiency. This discrepancy may lead students to overestimate their ability to communicate effectively in clinical encounters, potentially increasing the risk of communication errors or inappropriate reliance on their own language skills when professional interpreter services would be more appropriate. Future iterations of the module should emphasize this, and an assessment question on patient safety and appropriate use of interpreter services could be included.

Second, learner outcomes were assessed using two examination questions aligned with the module's learning objectives rather than standardized measures of Spanish proficiency or observed clinical communication. Although these questions assessed selected knowledge-based outcomes, they did not evaluate speaking, listening, pronunciation, or performance during patient interactions. Future studies should incorporate validated assessments of medical Spanish proficiency, learner confidence, and observed clinical communication through methods such as standardized patient encounters or objective structured clinical examinations. In addition, the content was not uniformly retained at the time of assessment. Several factors may have contributed, including the brief, one-time nature of the session, the one-month interval between the presentation and assessment, and competing curricular demands. Because the assessment was embedded within a summative course examination and was not specifically designed as a validated measure of the module, it cannot be determined whether incorrect responses reflected a lack of learning, forgetting, question interpretation, or other factors.

Third, students entered the session with varying levels of prior Spanish language exposure, which may have influenced learning outcomes and subsequent engagement with additional medical Spanish opportunities. Additionally, students who pursued subsequent Spanish electives may have had pre-existing interest in language learning or service with Spanish-speaking communities, which was not measured in this study. Future studies should consider collecting baseline language experience, learner motivation, and prior exposure to Spanish-speaking populations.

Fourth, this intervention was implemented at a single institution, and the findings may not be generalizable to other medical school settings with different curricular structures, student populations, or available resources. The module was developed and delivered by a medical librarian with formal academic training in Spanish language and culture rather than by a clinician or certified medical Spanish educator. Although content was informed by established educational resources and reviewed for accuracy, the module was intentionally designed as an introductory educational experience rather than comprehensive language instruction. Successful implementation at other institutions may benefit from collaboration among librarians, language educators, clinicians, interpreters, and community partners to ensure content accuracy, cultural relevance, and alignment with local needs.

Finally, delivery of this module requires some Spanish language knowledge. Although the presenter does not need to be fluent in Spanish, they should have sufficient proficiency to facilitate the game and dialogue activities and answer basic student questions. This requirement may present a barrier for institutions without faculty or staff who possess these skills. However, since the module is available as an open educational resource with detailed presenter notes, instructors with foundational Spanish knowledge may be able to implement it with minimal additional preparation. The module could also be adapted for team-teaching with a language instructor or bilingual clinician or modified to rely more heavily on the accompanying presenter notes and supplemental resources.

Despite these limitations, this intervention demonstrates that foundational concepts related to medical Spanish, cultural humility, and language-concordant care can be incorporated into existing medical curricula using minimal time and resources. Brief, adaptable educational interventions may serve as an accessible entry point for institutions seeking to introduce medical Spanish education and promote the continued development of communication skills necessary for equitable patient care. Future iterations could explore strategies to improve scalability, such as prerecorded audio, facilitator training materials, or co-teaching models.

In conclusion, as the number of Spanish-speaking patients in the United States continues to increase, medical schools play an important role in preparing future physicians to communicate effectively, safely, and respectfully with LEP patients. This study demonstrates that a brief, OER–based medical Spanish module can be integrated into a preclinical curriculum to introduce foundational concepts related to medical Spanish, cultural humility, patient safety, and language-concordant care. Although a single one-hour session cannot establish clinical language proficiency, it can increase awareness of language barriers, introduce strategies for effective communication, and encourage learners to pursue continued medical Spanish training.

The availability of adaptable, freely accessible educational materials may reduce barriers to implementation in institutions with limited curricular time, funding, or faculty expertise in medical Spanish. As an entry point within a longitudinal approach to language education, brief interventions such as this may complement more comprehensive curricula and support the development of physicians prepared to care for linguistically diverse populations. Scalable, OER-based approaches may provide a practical strategy for expanding medical Spanish education across the continuum of undergraduate medical training.

## Data Availability

The original contributions presented in the study are included in the article/Supplementary Material, further inquiries can be directed to the corresponding author.
