# Supplementary material for: Developing a one-shot medical Spanish module for medical students: an open educational resource approach
Source: Front Med (Lausanne). 2026 Sep 17;13:1957506. doi: 10.3389/fmed.2026.1957506 (PMC13627033; doi:10.3389/fmed.2026.1957506)
Supplement: Supplementary file 1 [file Datasheet1.pdf]

Title: Developing a One-Shot Medical Spanish Module for Medical Students: An Open Educational Resource Approach

Journal: Frontiers in Medicine

Author: Juli McCarroll

Affiliation: Western Michigan University Homer Stryker M.D. School of Medicine, Department of Medical Library, 1000 Oakland Drive, Kalamazoo, MI, United States of America

E-mail: juli.mccarroll@wmed.edu

MEDICAL SPANISH/ ESPAÑOL MÉDICO

Juli McCarroll, MA, MLIS, AHIP
Western Michigan University Homer Stryker M.D. School of Medicine
Department of Medical Library

CONFLICT OF INTEREST/ CONFLICTOS DE INTERÉS

none/ninguno

LICENSE

- Medical Spanish Module © 2026 by Juli McCarroll is licensed under a Creative Commons Attribution–NonCommercial 4.0 International License (CC BY-NC 4.0), which allows users to distribute, remix, adapt, and build upon the material in any medium or format, for noncommercial purposes.

- You must give appropriate credit, provide a link to the license, and indicate if changes were made.

- To view a copy of this license, visit https://creativecommons.org/licenses/by-nc/4.0/

LEARNING OBJECTIVES/ OBJETIVOS DE APRENDIZAJE

1. Describe four conditions that Latino patients may present with.
2. In Spanish, introduce yourself to a Spanish-speaking patient and ask how they're feeling.
3. Name two Spanish translation resources you can refer to if you need language assistance.

BIO/ ACERCA DE MÍ

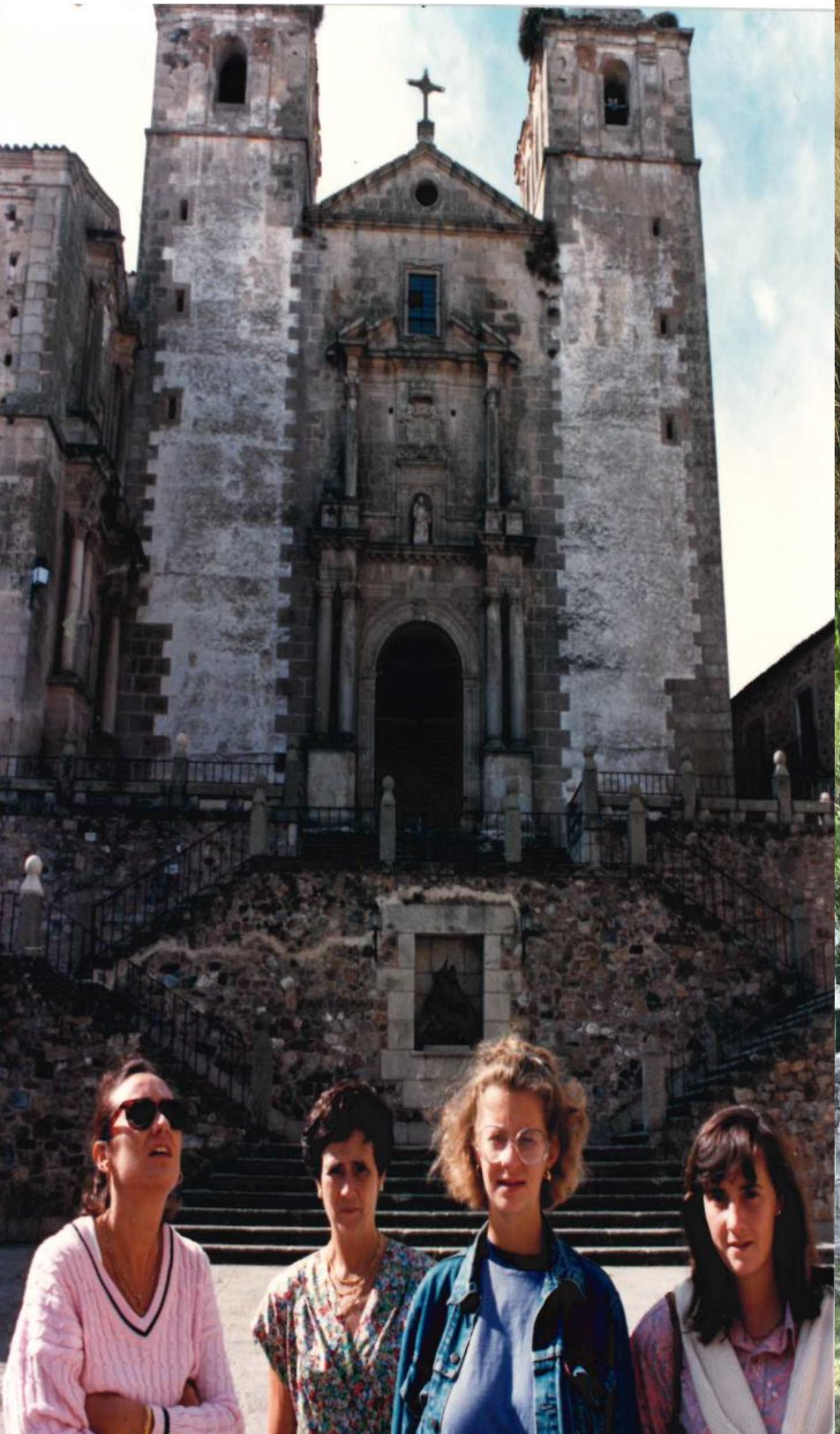

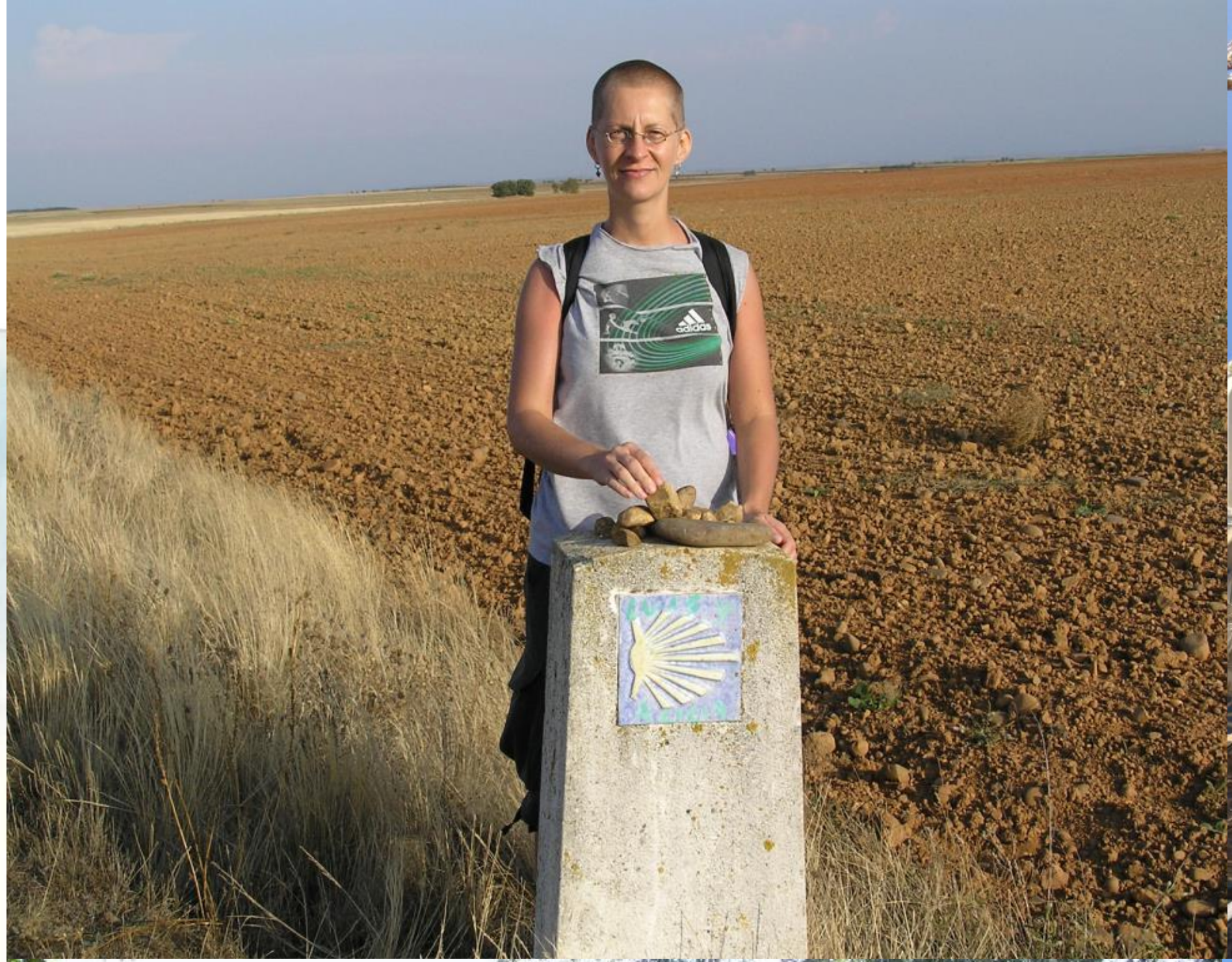

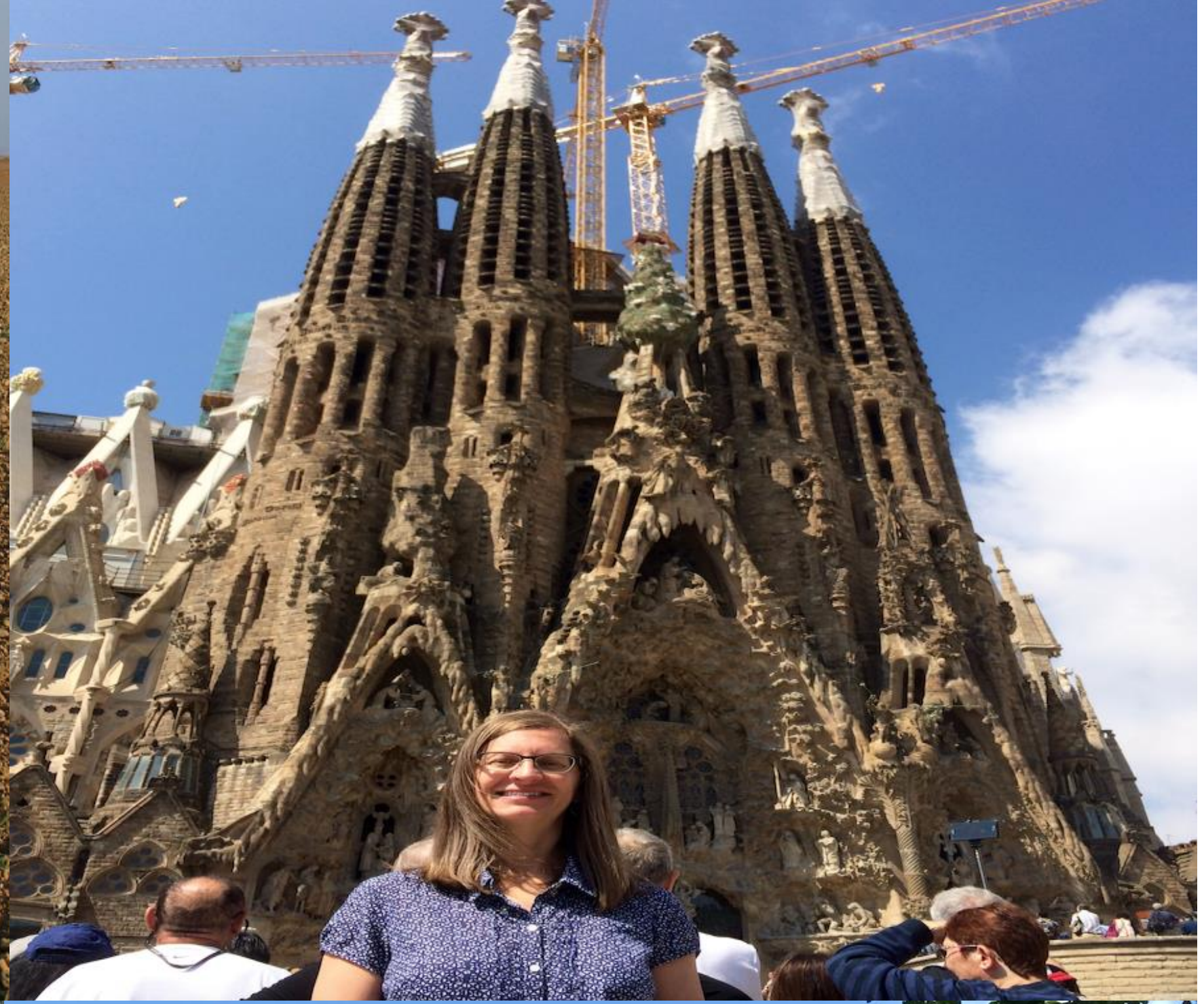

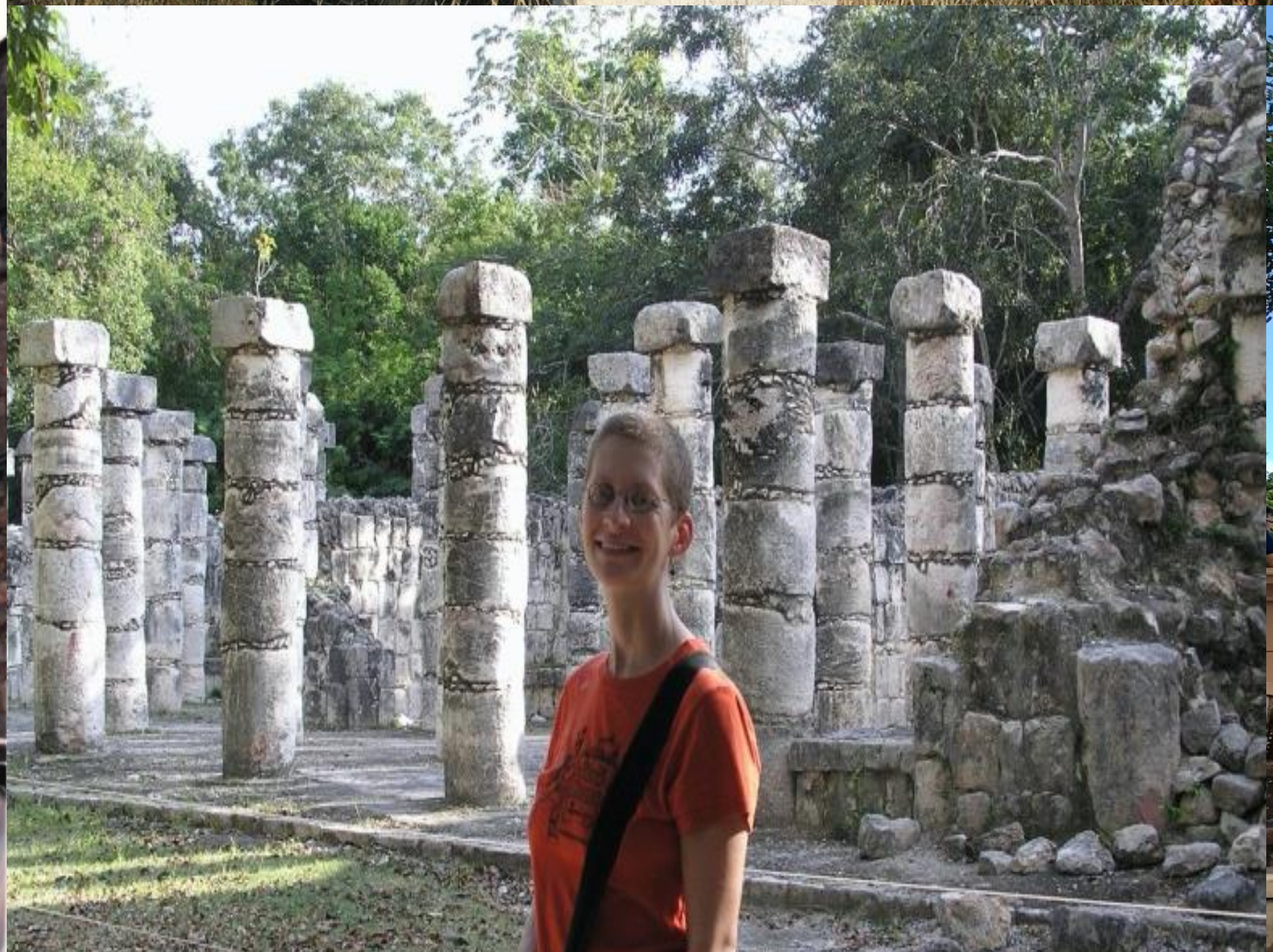

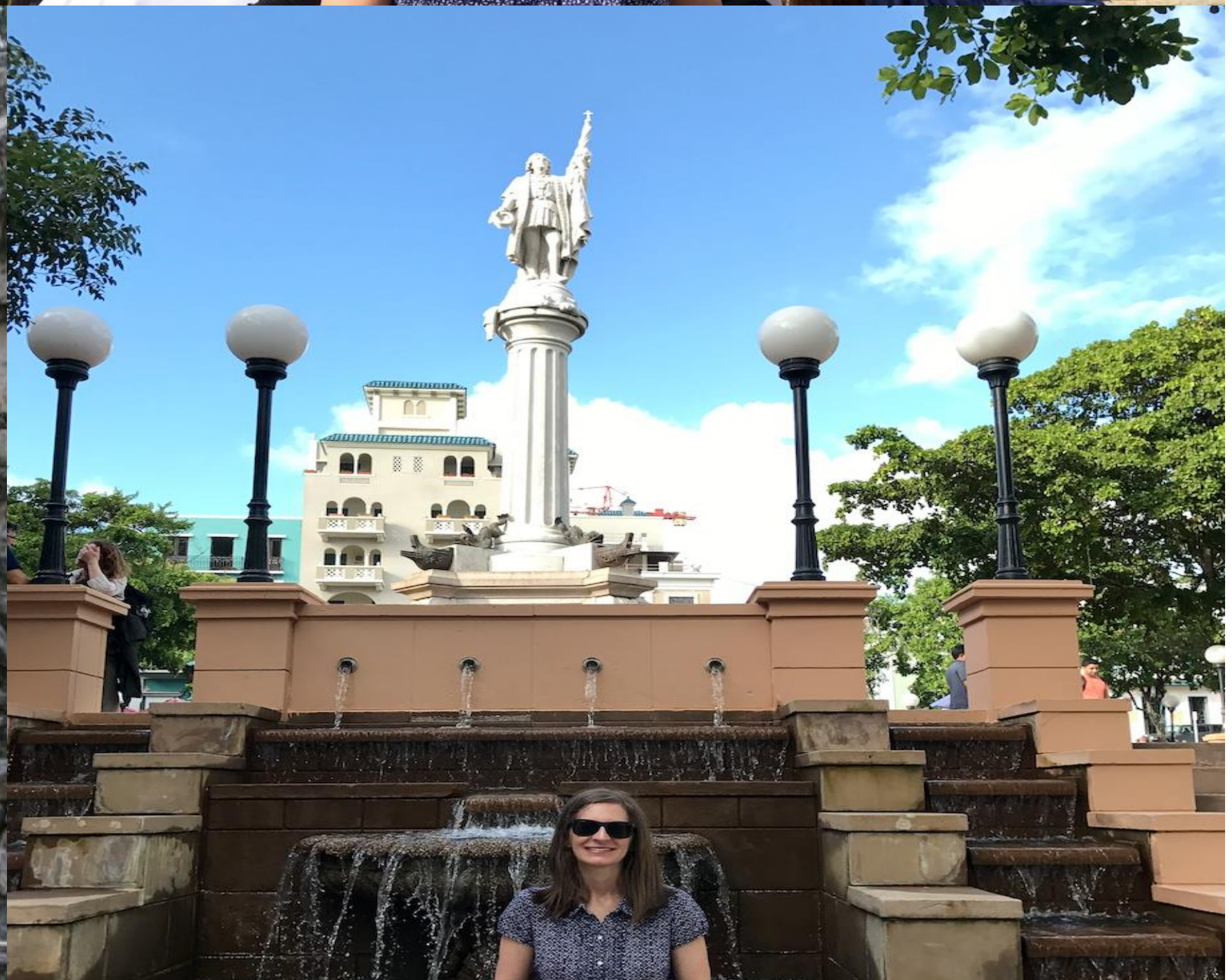

WHY LEARN ABOUT THIS?/ ¿POR QUÉ ESTUDIAR ESTO?

- LCME accreditation standards
- Language concordance
- Patient safety
- Cultural humility
- Practical communication skills
- Lifelong learning

¡LOTERÍA!

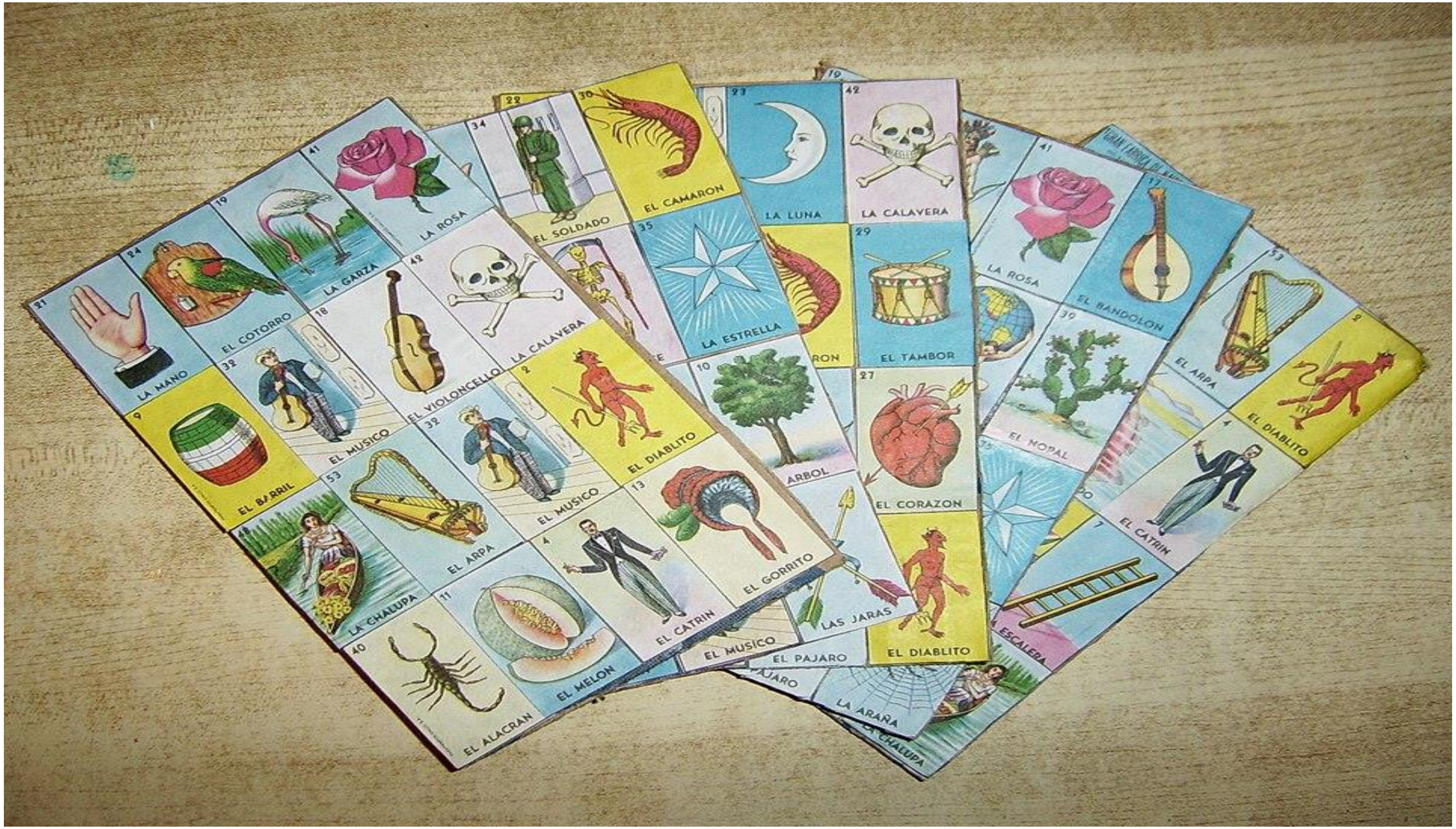

https://commons.wikimedia.org/w/index.php?curid=2064299

BODY PARTS/LAS PARTES DEL CUERPO

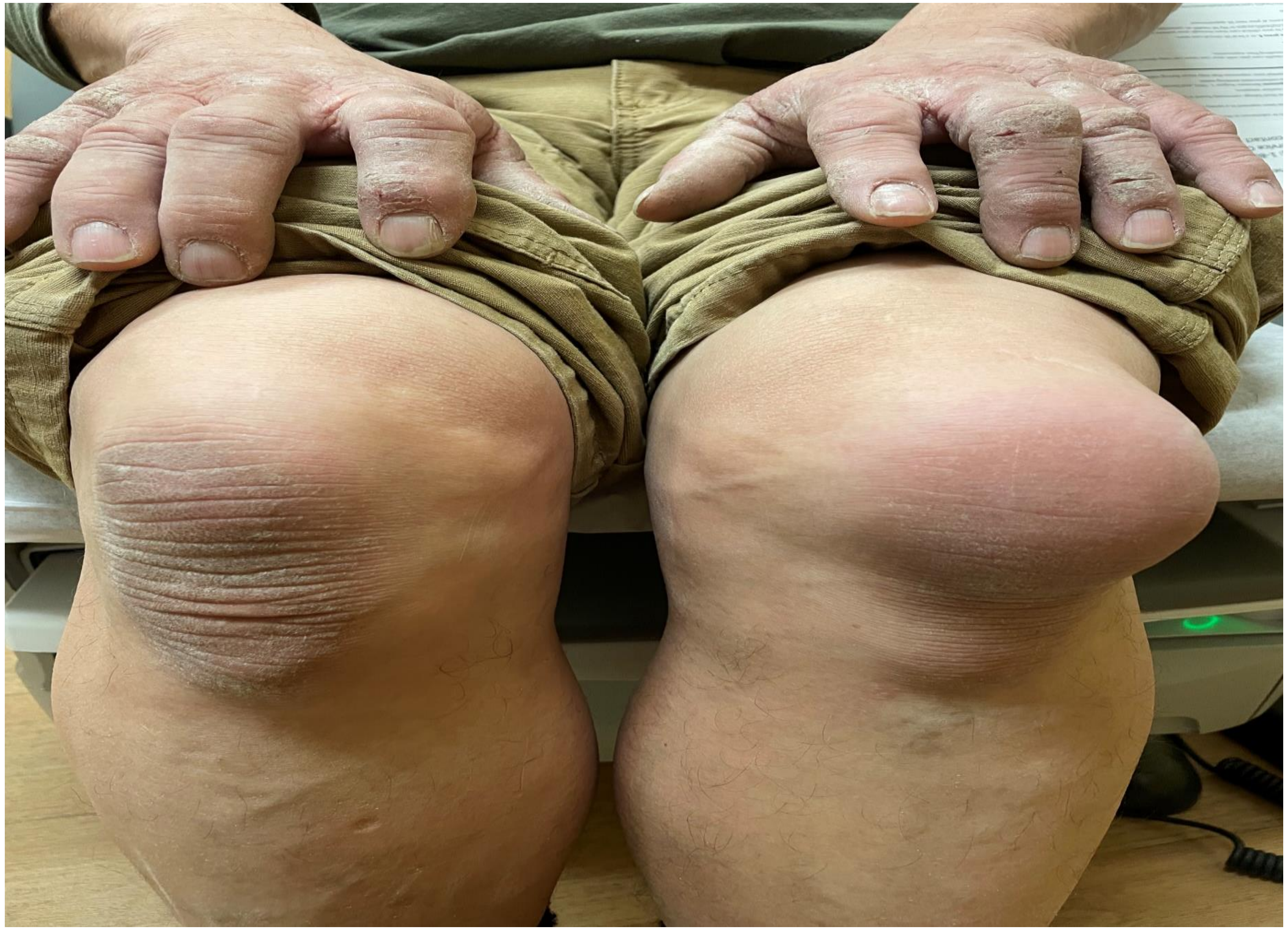

knees/las rodillas

BODY PARTS/LAS PARTES DEL CUERPO

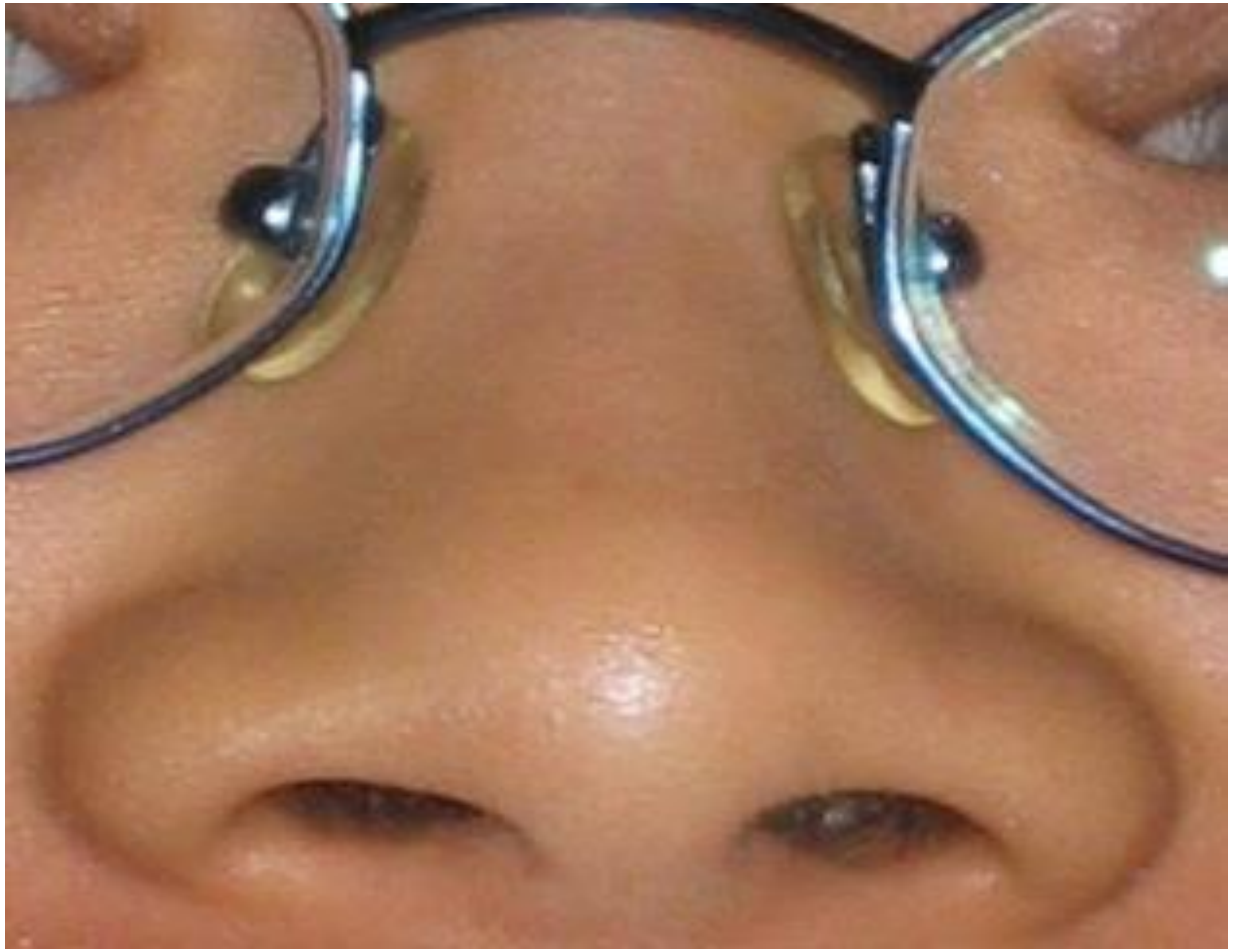

nose/el nariz

BODY PARTS/LAS PARTES DEL CUERPO

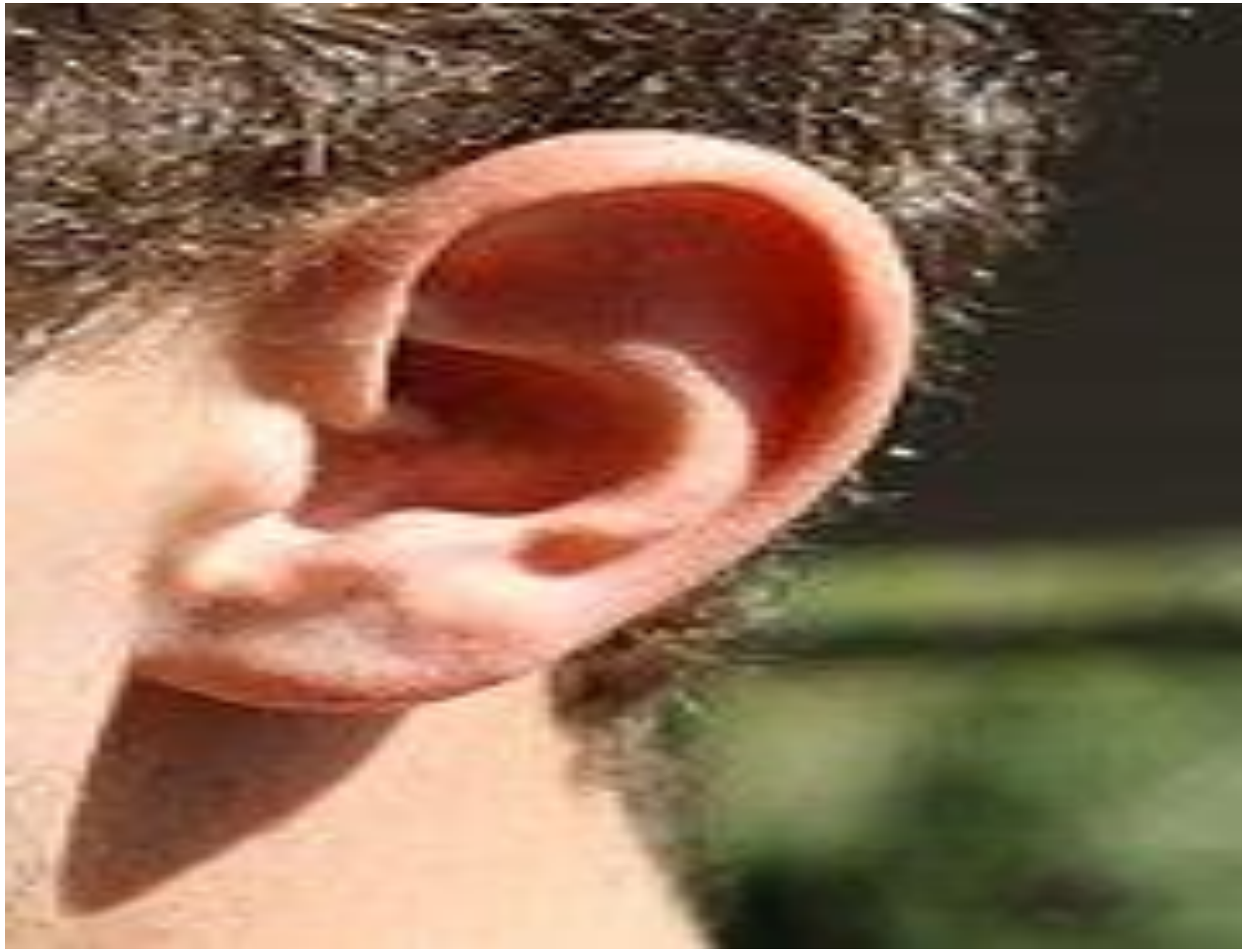

ear/el oído

BODY PARTS/LAS PARTES DEL CUERPO

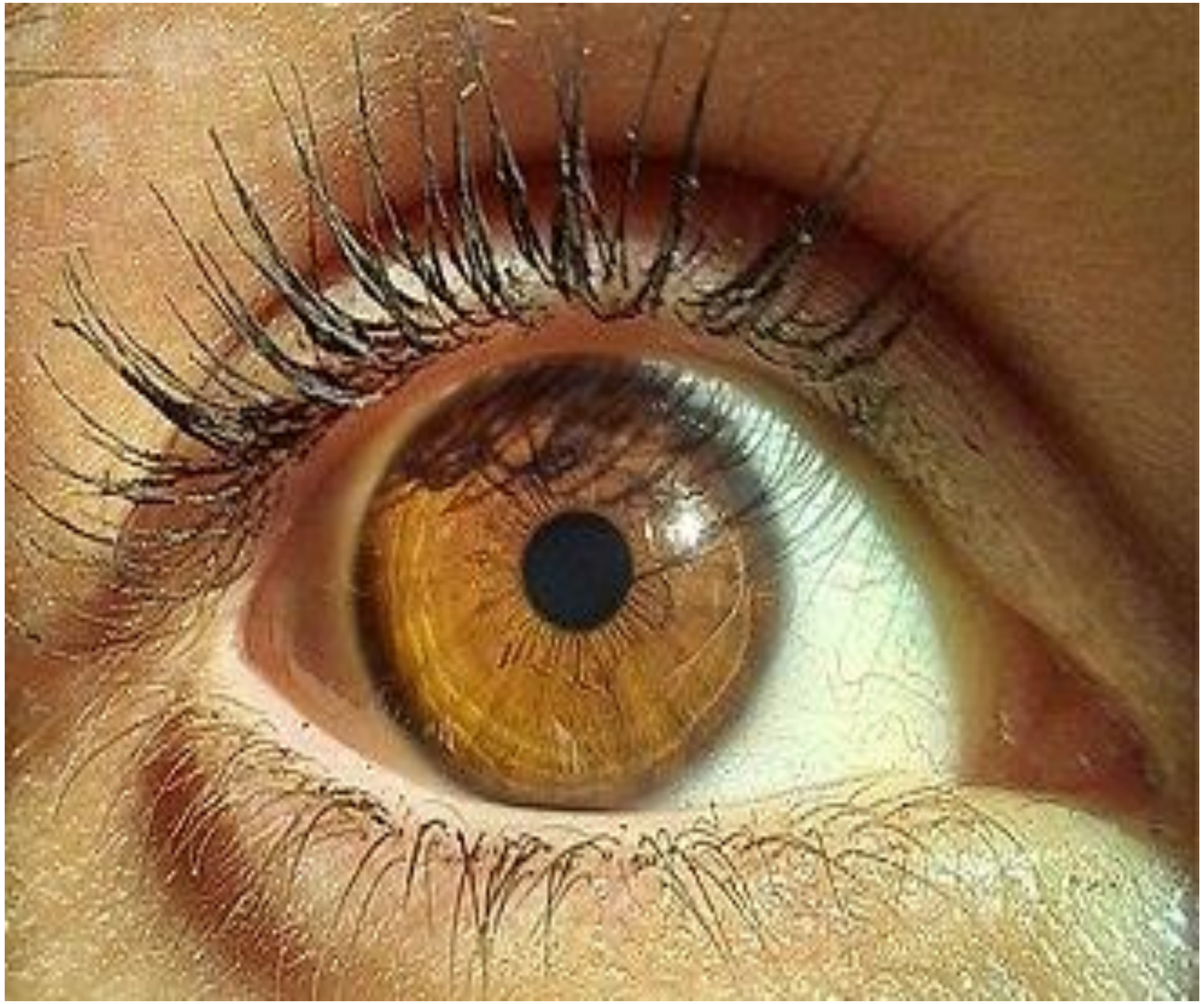

eye/el ojo

BODY PARTS/LAS PARTES DEL CUERPO

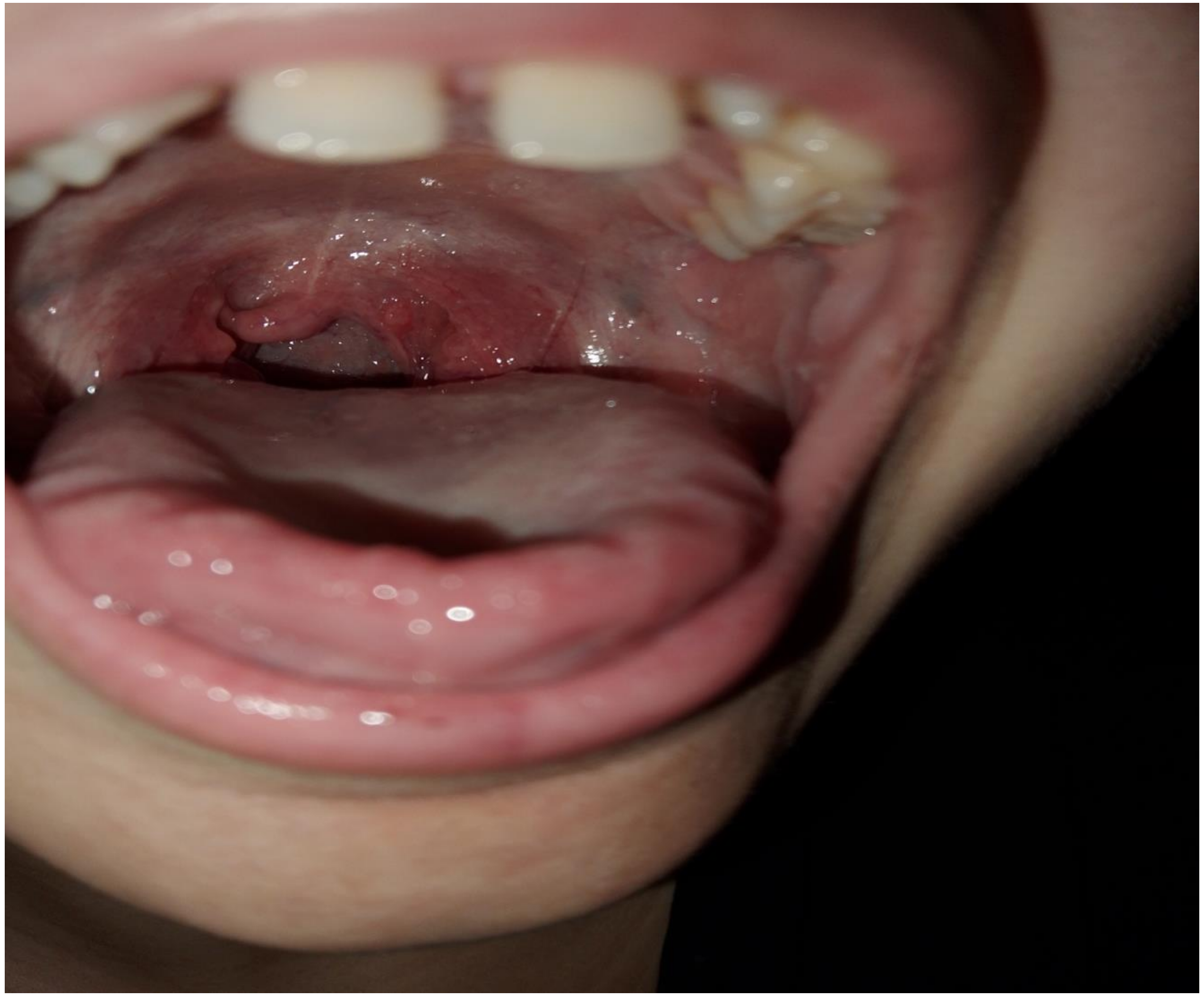

throat/la garganta

BODY PARTS/LAS PARTES DEL CUERPO

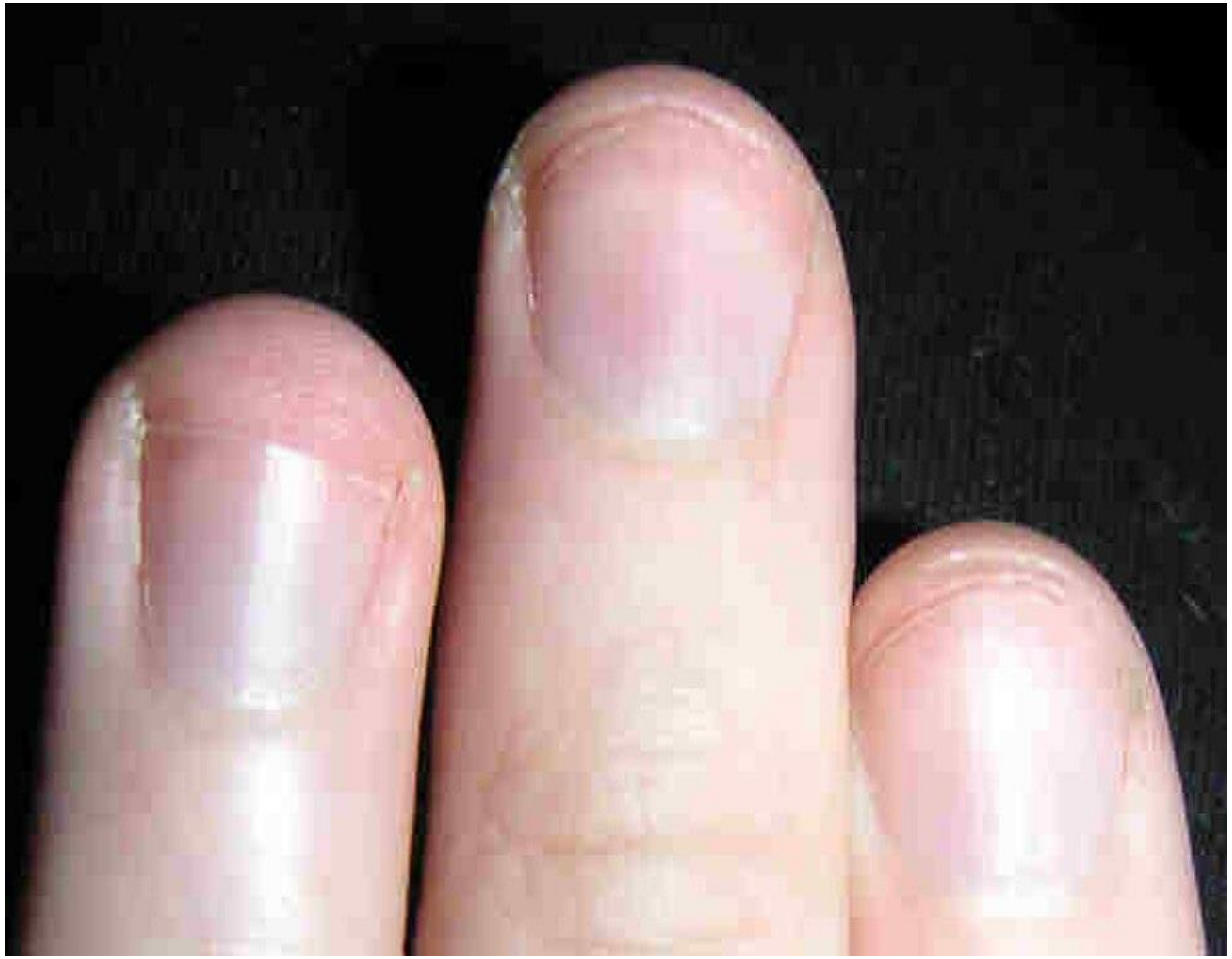

nails/las uñas

BODY PARTS/LAS PARTES DEL CUERPO

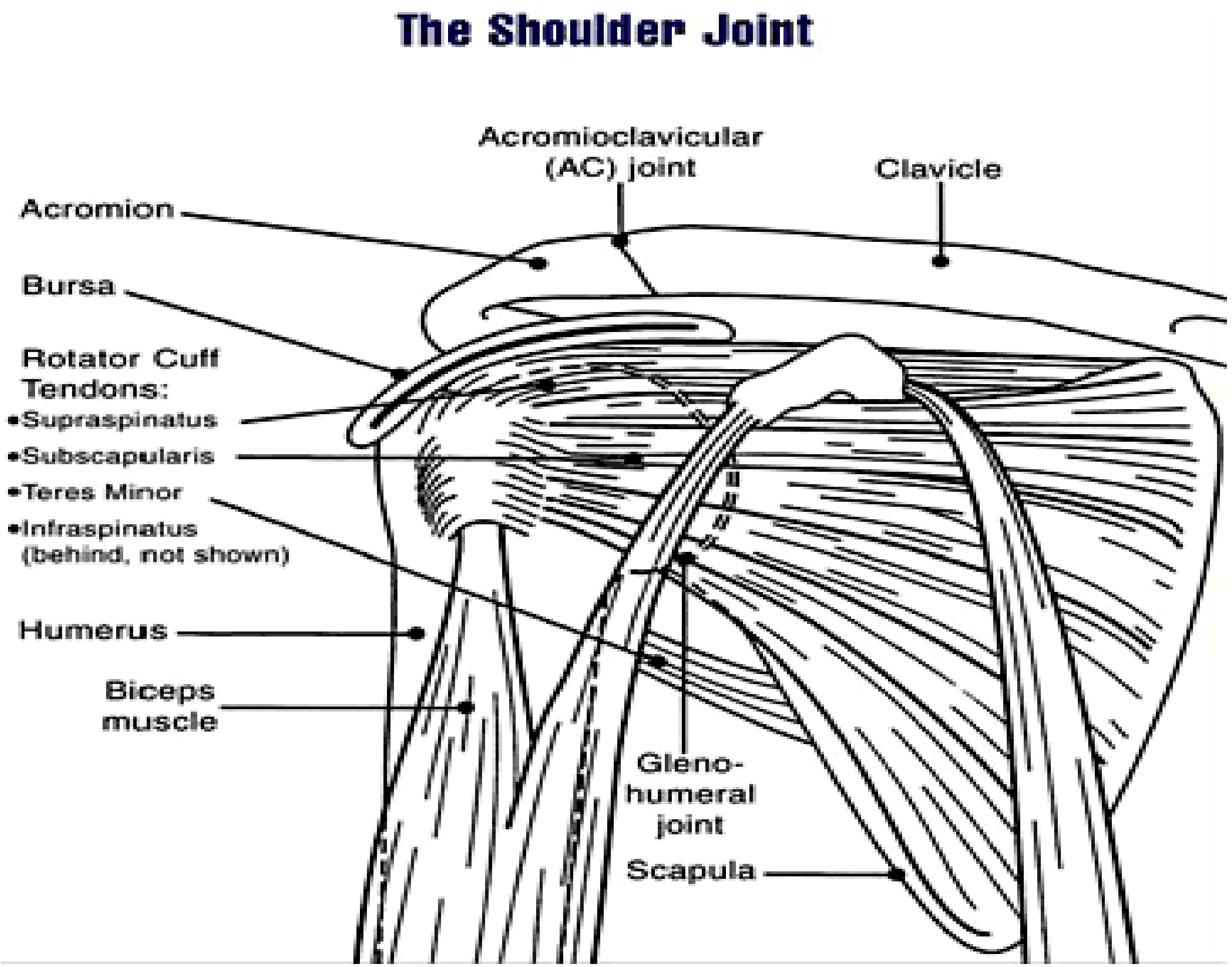

shoulder/el hombro

BODY PARTS/LAS PARTES DEL CUERPO

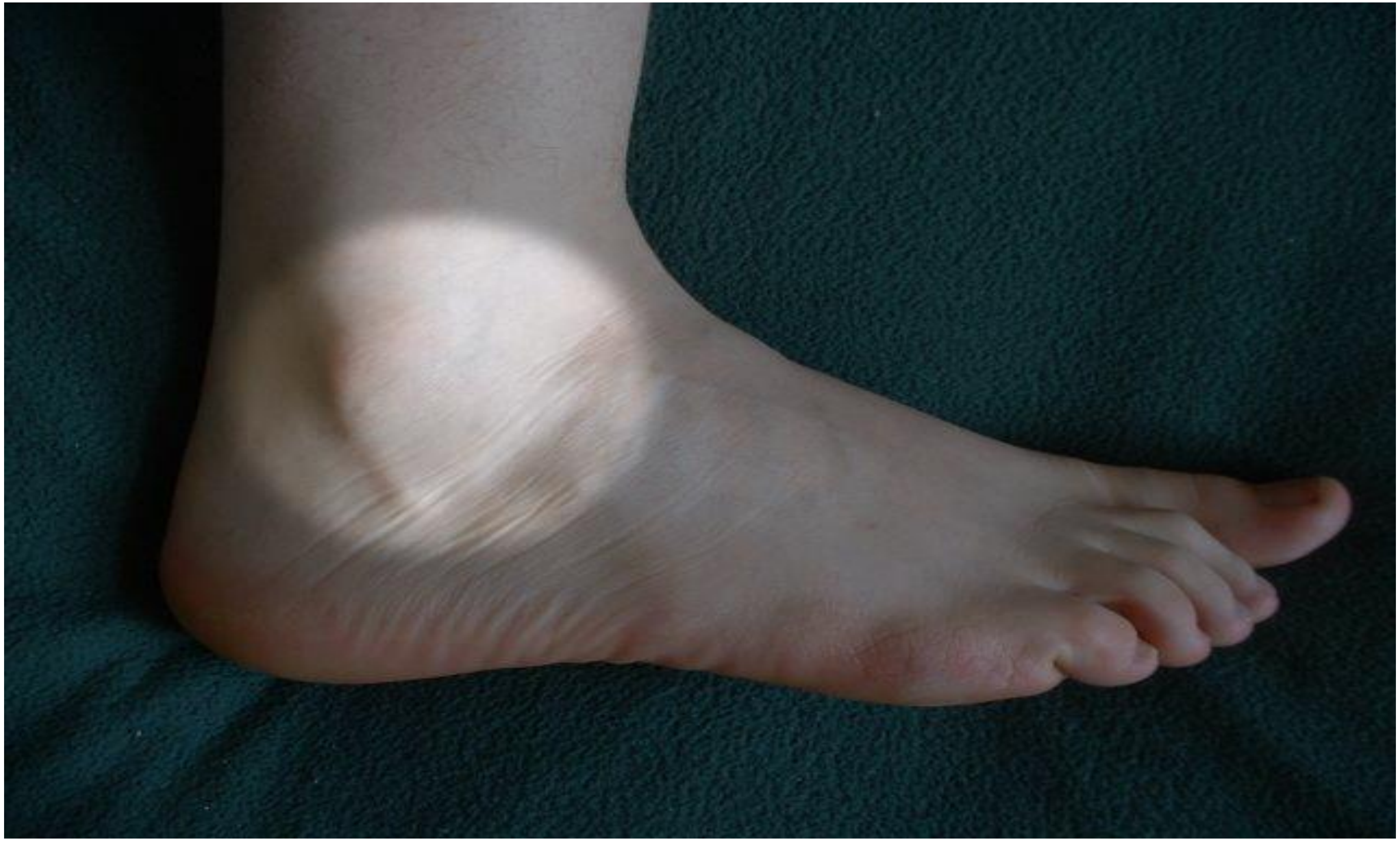

ankle/el tobillo

BODY PARTS/LAS PARTES DEL CUERPO

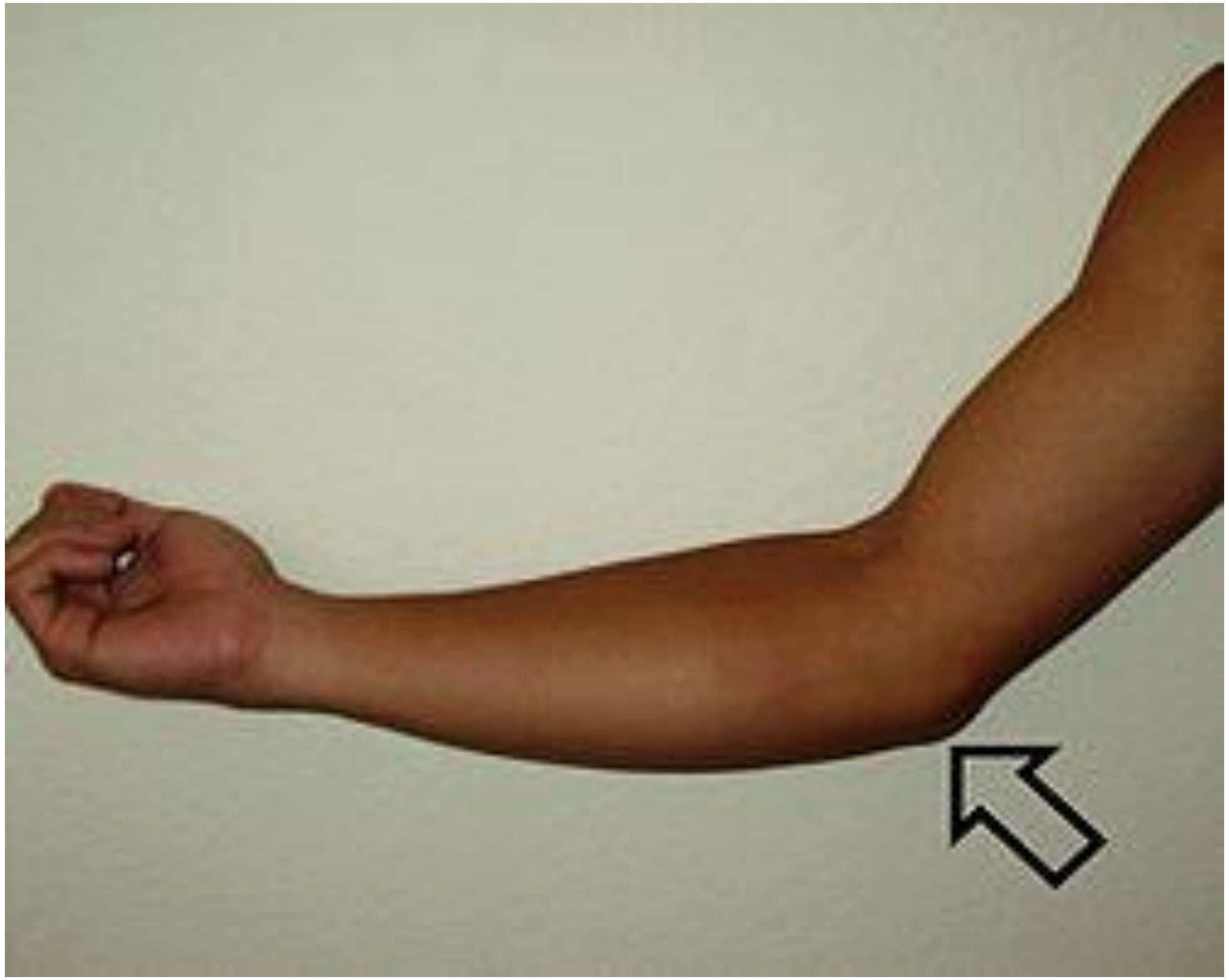

elbow/el codo

BODY PARTS/LAS PARTES DEL CUERPO

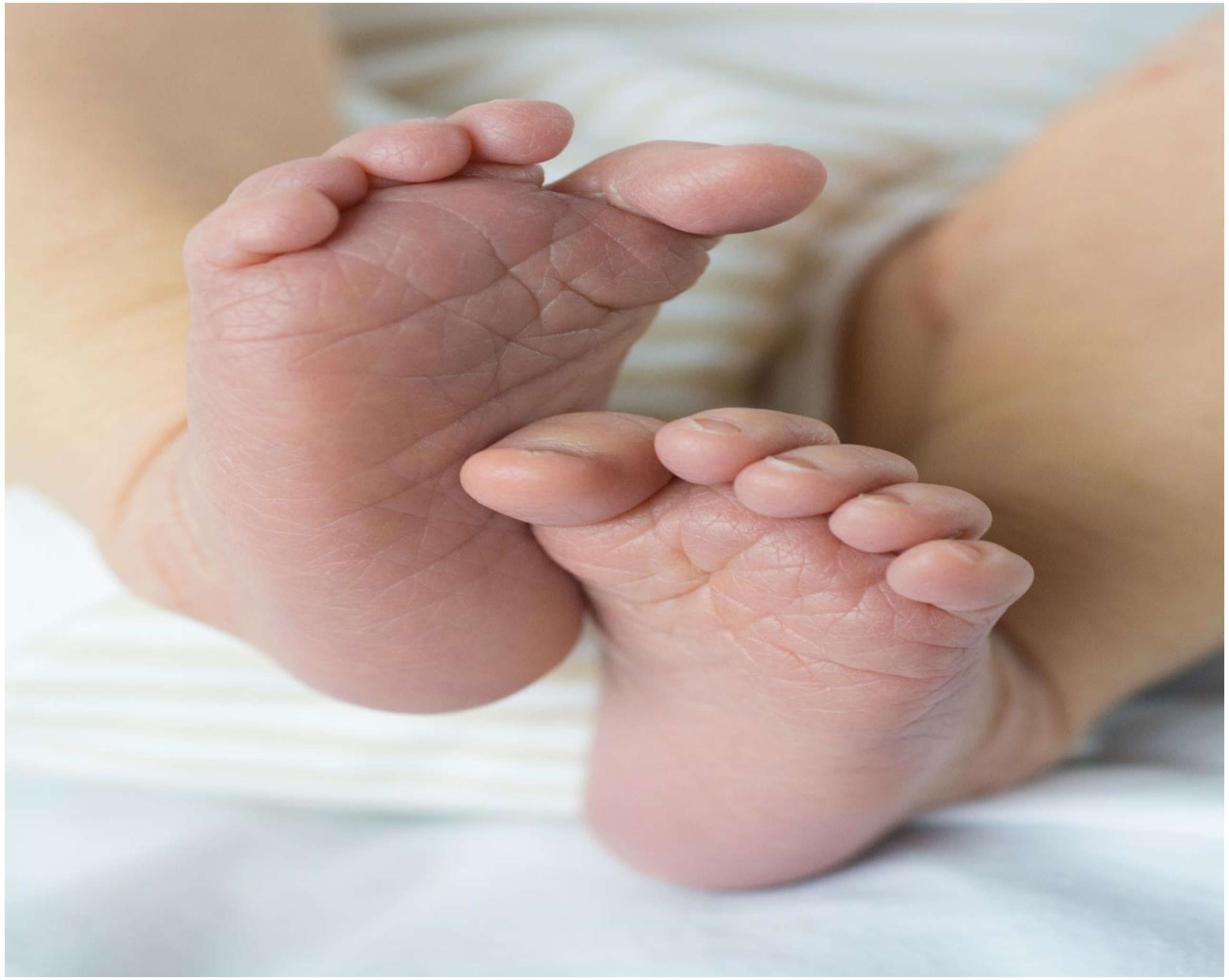

feet/los pies

BODY PARTS/LAS PARTES DEL CUERPO

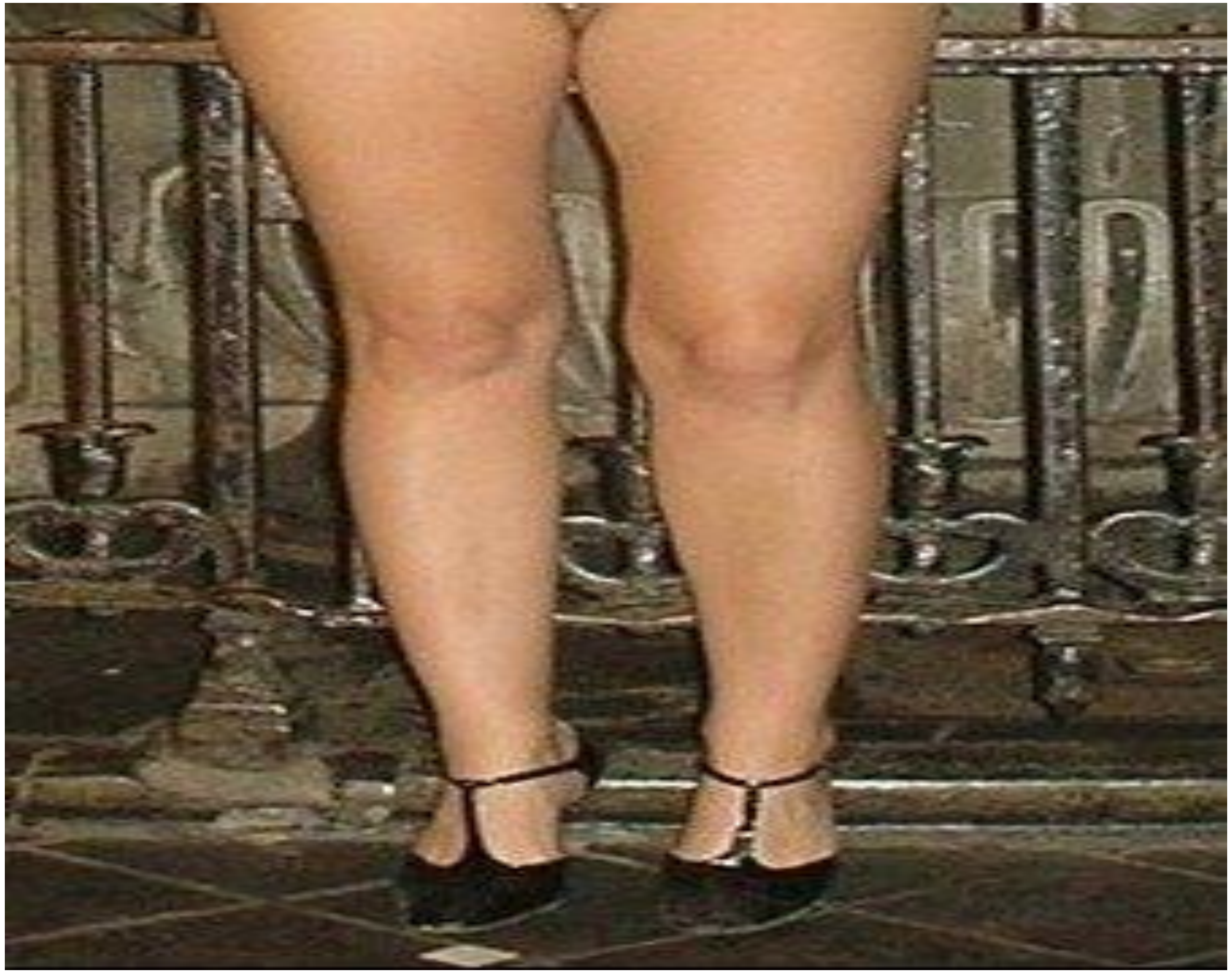

legs/las piernas

BODY PARTS/LAS PARTES DEL CUERPO

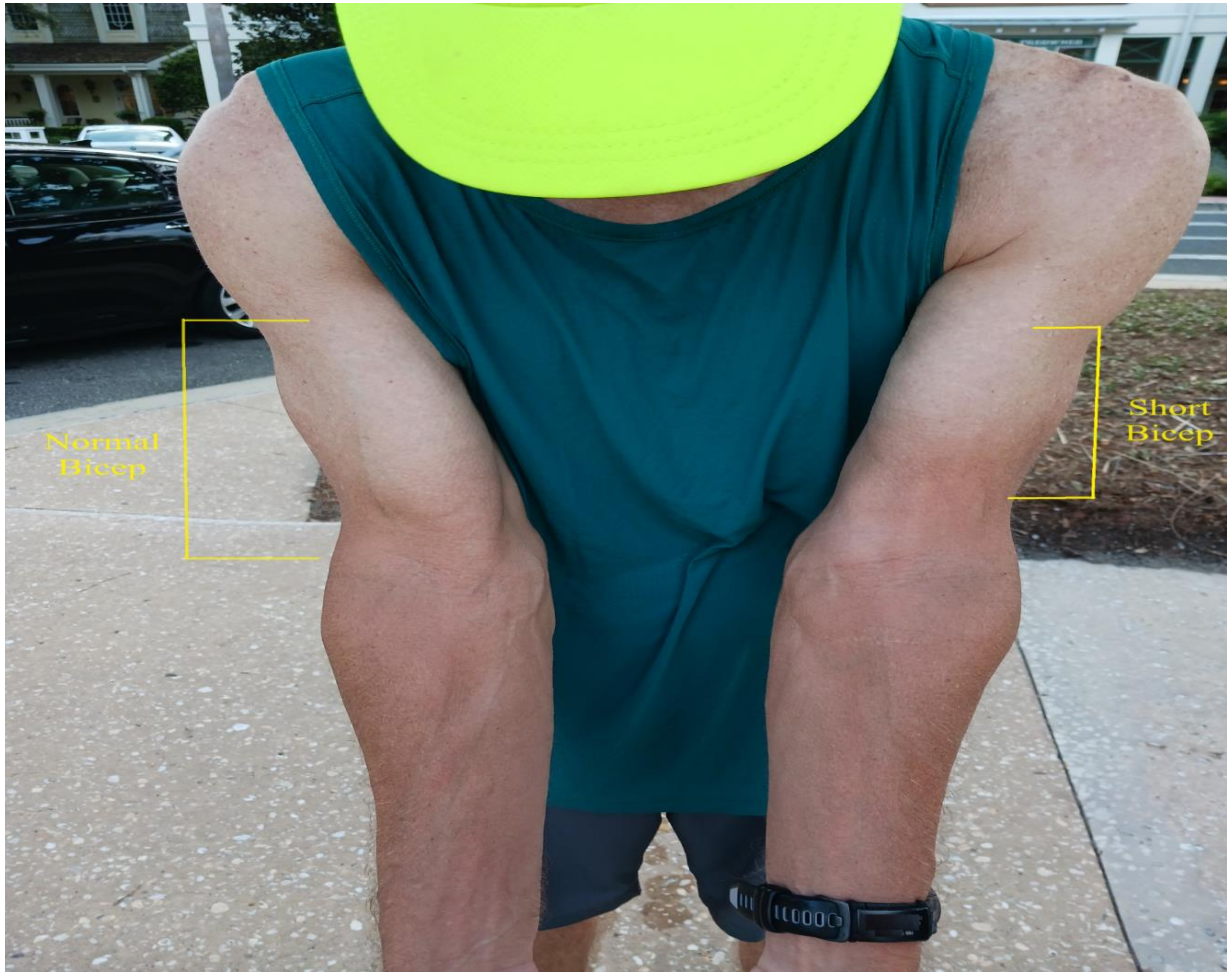

arms/los brazos

BODY PARTS/LAS PARTES DEL CUERPO

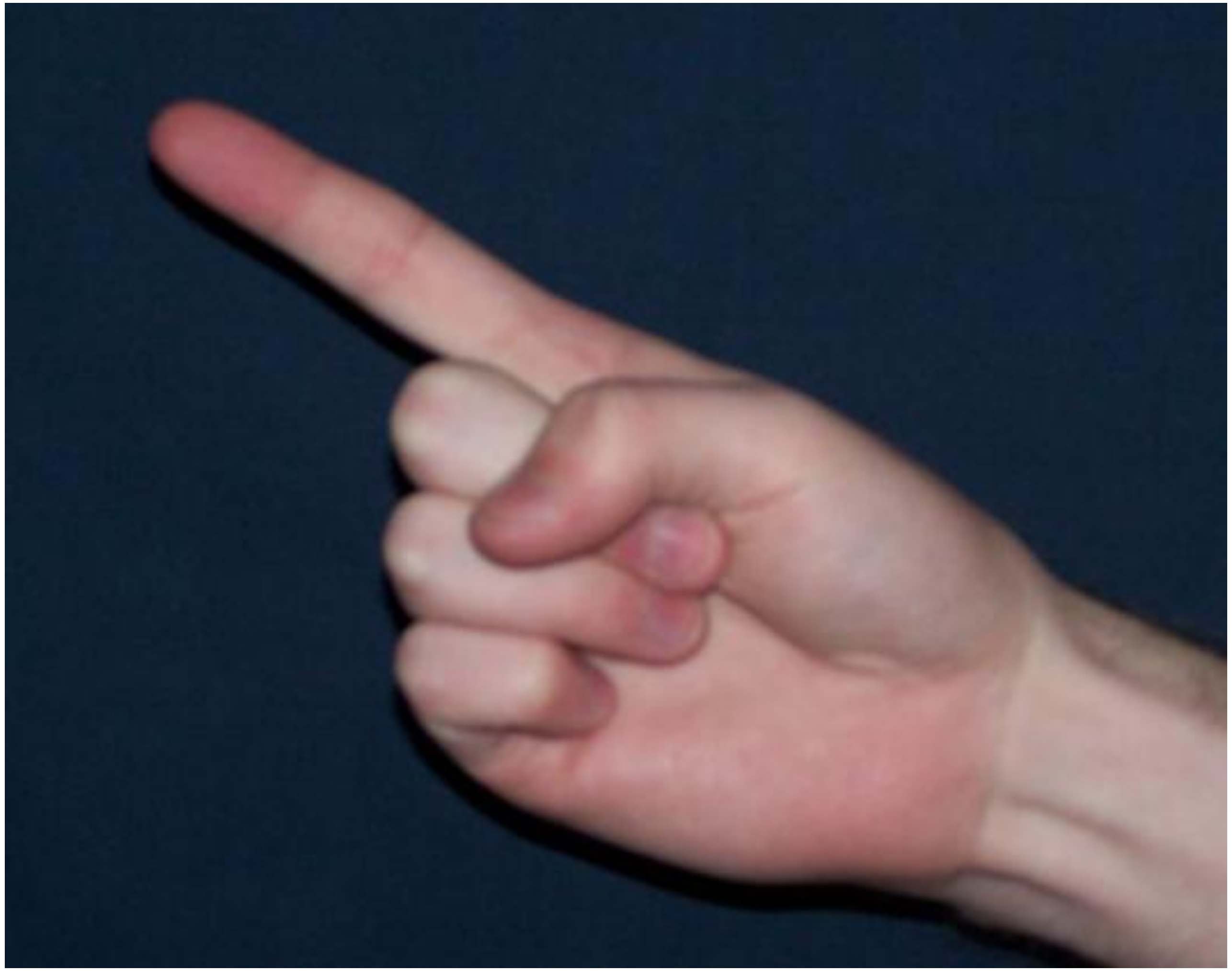

finger/el dedo

BODY PARTS/LAS PARTES DEL CUERPO

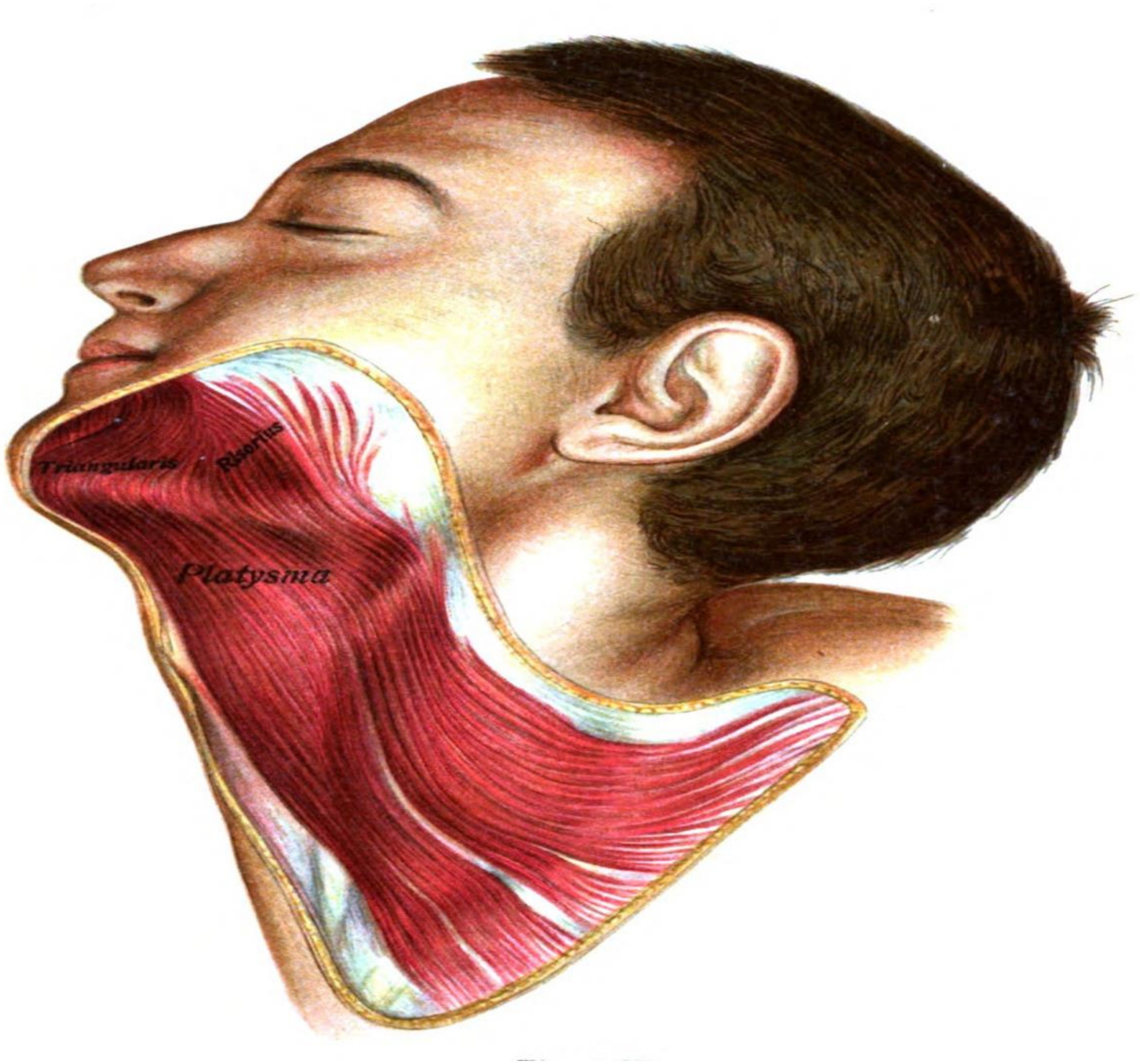

neck/el cuello

BODY PARTS/LAS PARTES DEL CUERPO

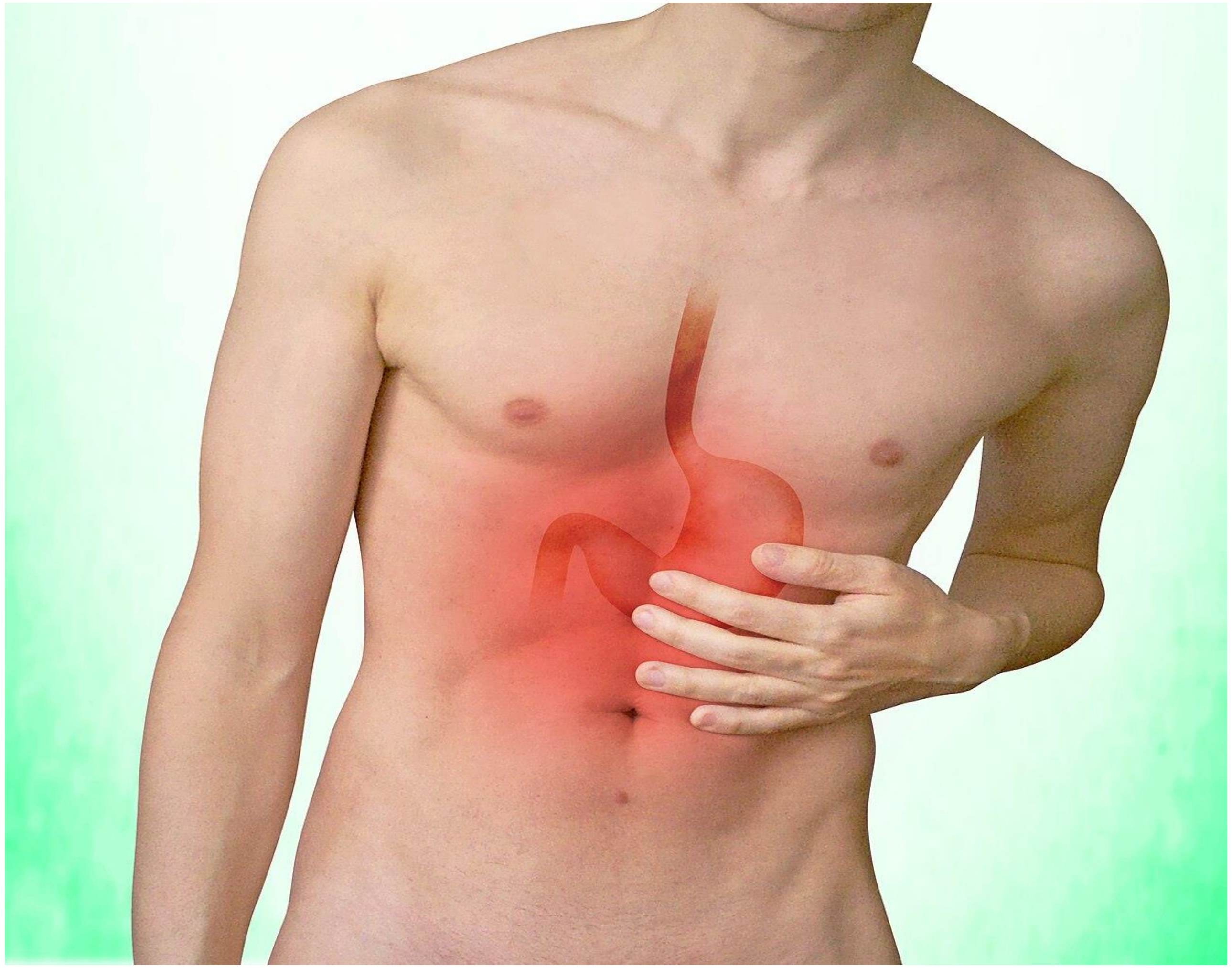

stomach/el estómago

BODY PARTS/LAS PARTES DEL CUERPO

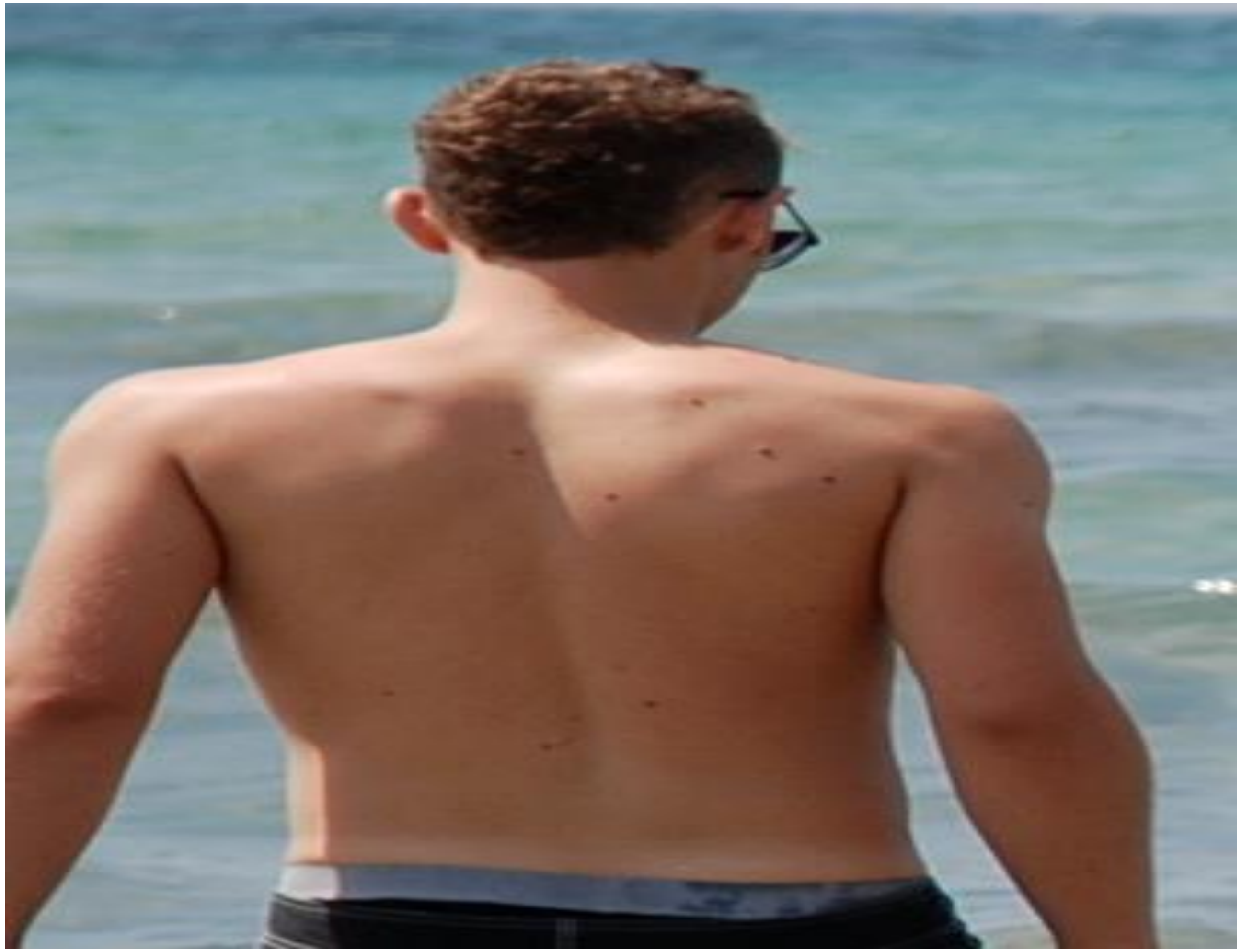

back/la espalda

AILMENTS/LAS DOLENCIAS

- Leading causes of death among Hispanics/Latinos:
  - Cancer
  - Heart Disease
  - Unintentional Injuries
  - Stroke
  - Diabetes
- Most common conditions:
  - Obesity
  - Hypertension
  - Diabetes

https://www.hitn.org/state-of-health-among-hispanics-in-the-us-in-2022/
https://minorityhealth.hhs.gov/hispaniclatino-health

NERVES/LOS NERVIOS

- Folk illness that can present like PTSD, anxiety, depression, stress, schizophrenia
- Can develop in children or adults
- Causes:
  - stress, fright, family/spouse issues, getting angry
- Symptoms
  - depression, worry, impatience, headache, toothache
- Treatment:
  - chamomile tea, going to a traditional healer or pharmacy

https://pubmed.ncbi.nlm.nih.gov/14510097/, https://pubmed.ncbi.nlm.nih.gov/22270268/, https://pubmed.ncbi.nlm.nih.gov/30612305/ , https://pubmed.ncbi.nlm.nih.gov/18535889/

INDIGESTION/EL EMPACHO

- Culturally-bound folk illness with many possible causes, symptoms, cures
- Presents like severe indigestion, dyspepsia, gastroparesis
- Causes:
  - eating too much/too fast/spoiled or greasy food/when you're not hungry/on the run, eating and smoking, drinking alcohol/caffeine, swallowing gum
- Symptoms:
  - stomachache, bloating, cramps, diarrhea, vomiting, nausea, gas, dry mouth, tiredness, headache, and perspiration

Treatment:

- drink mint, green, and chamomile tea, massage the stomach and back

- Prevention:
  - eat less food more often, start the meal with soup or salad

https://pubmed.ncbi.nlm.nih.gov/23684719/, https://www.pinterest.com/pin/699535754611664565/, https://www.ecoceutics.com/respuestas-dsalud/salud/empacho , https://pubmed.ncbi.nlm.nih.gov/34106187/

FRIGHT/EL SUSTO

- Culturally-bound illness that can present like depression, PTSD, panic attack
- Like nervios, can be used to describe schizophrenia
- Cause:
  - frightening event or experience that attacks the spirit and causes the soul to leave the body
- Symptoms:
  - insomnia, lethargy, lack of participation in or enjoyment of activities, detachment from life and people, nervousness, anorexia, fever, diarrhea
- Treatment:
  - ceremonies with a traditional healer
- Talk to the patient about the event that caused onset

https://pubmed.ncbi.nlm.nih.gov/22270268/ , https://pubmed.ncbi.nlm.nih.gov/36991563/ , https://pubmed.ncbi.nlm.nih.gov/22539060/ , https://pubmed.ncbi.nlm.nih.gov/29541008/ , https://pubmed.ncbi.nlm.nih.gov/35243566/ , https://pubmed.ncbi.nlm.nih.gov/18535889/

QUARANTINE/LA CUARENTENA

- 40-day post-partum recuperation period
- Mother is given a lot of support
- Makes breastfeeding more convenient
- DOs:
  - Stay indoors, eat warm/healthy/homemade foods
- DONTs:
  - work, engage in sexual activity, eat pre-packaged/unhealthy/greasy/raw/cold/green/spicy/sweet/acidic foods
- Mothers may be superstitious and not want to violate la cuarentena

https://pubmed.ncbi.nlm.nih.gov/22092447/, https://pmc.ncbi.nlm.nih.gov/articles/PMC8256751/?utm_source=chatgpt.com

HOME REMEDIES/LOS REMEDIOS CASEROS

- 65% age 45 and older believe herbal remedies and supplements are effective
- 62% use them occasionally, 27% at least once/week, 16% daily
- Use is higher among those not born in the U.S.
- 70% did not consult a doctor before starting home remedies
- Some use home remedies instead of prescribed medication
- Other studies look at medicinal plant use for inflammatory and neuropathic pain, remedies to prevent and mitigate COVID-19 symptoms

https://assets.aarp.org/rgcenter/health/segunda_herbals.pdf, https://pubmed.ncbi.nlm.nih.gov/40415521/, https://pubmed.ncbi.nlm.nih.gov/33923101/

HOME REMEDIES/LOS REMEDIOS CASEROS

- Chamomile/la manzanilla- colic, upset stomach, nausea, cramps, vomiting
- Aloe/la sábila-burns, sores, acne, cuts
- Rue/la ruda-earache
- Anise/el anís-fright
- Mint/la hierbabuena-cold, colic, upset stomach, nerves, intestinal issues
- Wormwood/el ajenjo-gas, colic, diarrhea, nausea
- Orange tree leaf tea/el té de naranjo-cramps, nerves, insomnia
- Basil/la albahaca-fright
- Oregano/el orégano-flu, cold, sore throat, congestion
- Garlic/el ajo-stomachache, insomnia, insect bite, arthritis, earache
- Vick's VapoRub-shortness of breath, laryngitis, sinuses, cold, flu

https://pubmed.ncbi.nlm.nih.gov/30860961/, https://https://pubmed.ncbi.nlm.nih.gov/24030377/, https://pubmed.ncbi.nlm.nih.gov/33923101/, https://pubmed.ncbi.nlm.nih.gov/40415521/

VACCINES/LAS VACUNAS

- Overall, Latinos have lower vaccine rates than the overall population
- Use varies depending on location, employment status, income
- Misinformation, patient-physician language discordance, limited health literacy in English, limited access to primary care, high cost of care, lack of health insurance, inflexible work schedules, immigration fears, transportation issues, lack of Latino-specific outreach, cultural beliefs and perceptions of vaccine necessity, lower trust in the healthcare system due to prior negative experiences may contribute to lower rates
- HHS data shows Latinos got the flu vaccine 23% less often
- Latinos age 65 and older got the pneumococcal vaccine 35% less often
- Latinos are 25% less likely to have received age-appropriate vaccines
- Latino children born in 2020 were 4% less likely to be fully vaccinated by age 2

https://minorityhealth.hhs.gov/immunizations-and-hispaniclatino-americans, https://pubmed.ncbi.nlm.nih.gov/35782479/, https://pubmed.ncbi.nlm.nih.gov/34140009/

MATCHING EXERCISE

- 1. Causes: stress, fright, family/spouse issues, getting angry
  Symptoms: depression, worry, impatience, headache, toothache
  Treatment: manzanilla tea, going to a traditional healer or pharmacy
- 2. Causes: eating too much/too fast/spoiled or greasy food/when you're not hungry/on the run, eating and smoking, drinking alcohol/caffeine, swallowing gum
  Symptoms: stomachache, bloating, cramps, diarrhea, vomiting, nausea, gas, dry mouth, tiredness, headache, perspiration
- 3. Cause: frightening event or experience that attacks the spirit and causes the soul to leave the body
  Symptoms: insomnia, lethargy, lack of participation in or enjoyment of activities, detachment from life and people, nervousness, anorexia, fever, diarrhea
- 4. Latinos are 1.7 times more likely to have it and 1.4 times more likely to die from it
- 5. 40-day post-partum recuperation period to bond with baby and heal physically and emotionally
- 6. Used to treat colic, upset stomach, nausea, cramps, vomiting, sore throat

MATCHING EXERCISE

A. el empacho

B. la manzanilla

C. la cuarentena

D. la diabetes

E. el susto

F. los nervios

MATCHING EXERCISE ANSWERS

1. Causes: stress, fright, family/spouse issues, getting angry
Symptoms: depression, worry, impatience, headache, toothache
Treatment: manzanilla tea, going to a traditional healer or pharmacy

F-los nervios

2. Causes: eating too much or too fast/spoiled or greasy food/when you're not hungry/on the run, eating and smoking, drinking alcohol/caffeine, swallowing gum
Symptoms: stomachache, bloating, cramps, diarrhea, vomiting, nausea, gas, dry mouth, tiredness, headache, perspiration

A-el empacho

3. Cause: frightening event or experience that attacks the spirit and causes the soul to leave the body
Symptoms: insomnia, lethargy, lack of participation in or enjoyment of activities, detachment from life and people, nervousness, anorexia, fever, diarrhea

E-el susto

4. Latinos are 1.7 times more likely to have it and 1.4 times more likely to die from it

D-la diabetes

5. 40-day post-partum recuperation period to bond with baby and heal physically and emotionally

C-la cuarentena

6. Used to treat colic, upset stomach, nausea, cramps, vomiting, sore throat

B-la manzanilla

DIALOGUE/DIÁLOGO

A: Good morning, sir. Good morning, ma'am. Good morning, miss.

Buenos días, señor. Buenos días, señora. Buenos días, señorita.

A: My name is Dr. _____.

Soy el doctor __/la doctora __. Me llamo Dr. __/Dra. _

A: What's your name?

¿Cómo se llama Usted (Ud.)?

B: I'm ____.

Soy ____. Me llamo ___.

A: Nice to meet you. How are you feeling?

Mucho gusto. ¿Cómo se siente?

DIALOGUE/DIÁLOGO

B: I don't feel good. I feel awful.

No me siento bien. Me siento muy mal.

A: Are you in any pain?

¿Tiene algún dolor?

B: Yes, my ear hurts. Yes, my head hurts. Yes, my knee hurts. Yes, my finger hurts.

Sí, me duele el oído. Sí, me duele la cabeza. Sí, me duele la rodilla. Sí, me duele el dedo.

A: Let me take a look.

Déjeme verlo. Déjeme verla. Déjeme verla. Déjeme verlo.

TRANSLATION RESOURCES/ RECURSOS DE TRADUCCIÓN

Google Translate

SpanishDict

Canopy Speak

NEXT STEPS/ PRÓXIMOS PASOS

- Check out the references and reading list.
- Use the translation websites and apps to practice..
- Get involved in the community.
- Enroll in a Spanish elective offered by the library.
- Have fun with the language!

LEARNING OBJECTIVES/ OBJETIVOS DE APRENDIZAJE

1. Describe four conditions that Latino patients may present with.
2. In Spanish, introduce yourself to a Spanish-speaking patient and ask how they're feeling.
3. Name two Spanish translation resources you can refer to if you need language assistance.

REFERENCES/READING LIST

Amirehsani KA, Hu J, Wallace DC, McCoy TP. Herbal/plant remedies and supplements used by Hispanics/Latinxs for diabetes: Source of functional foods?. *Sci Diabetes Self Manag Care*. 2021;47(1):94-104. doi:10.1111/j.1552-6909.2011.01298.x

Anderson G. Usage of herbal supplements and remedies among Hispanics 45+. American Association of Retired Persons. https://assets.aarp.org/rgcenter/health/segunda_herbals.pdf. Published March 2010. Accessed February 25, 2026.

Baer R, Weller S, Alba-García J, Glazer M, Trotter II R, Pachter L et al. A cross-cultural approach to the study of the folk illness nervios: Methodological advances in the cross-cultural study of mental health. *Cult Med Psychiatry*. 2003;27:315-337. doi 10.1023/A:1025351231862

Barcelo J, Blair E, Crespo R, Shah S, Junquera P, Matejka A. A Hispanic female presenting with the cultural syndrome "El Salto" as a psychosomatic symptom of anxiety. *Cureus*. 2025;17(9):e92861. Published 2025 Sep 21. doi:10.7759/cureus.92861

REFERENCES/READING LIST

CanopySpeak. Canopy. https://info.withcanopy.com/canopy-speak. Accessed February 25, 2026.

Cultural insights: Communicating with Hispanics/Latinos. Centers for Disease Control and Prevention. https://stacks.cdc.gov/view/cdc/13183. Published 2012. Accessed February 25, 2026.

Dejbord Sawan P. Beginning Medical Spanish: Oral Proficiency and Cultural Humility 1st ed. Routledge; 2020. https://www.routledge.com/Beginning-Medical-Spanish-Oral-Proficiency-and-Cultural-Humility/DejbordSawan/p/book/9780367322434.

Diabetes and Hispanic/Latino Americans. U.S. Department of Health and Human Services Office of Minority Health. https://minorityhealth.hhs.gov/diabetes-and-hispaniclatino-americans. Updated January 2026.

Dura-Vila G, Hodes M. Cross-cultural study of idioms of distress among Spanish nationals and Hispanic American migrants: Susto, nervios and ataque de nervios. *Soc Psychiatry Psychiatr Epidemiol*. 2012;47(10):1627-1637. doi:10.1007/s00127-011-0468-3.

REFERENCES/READING LIST

Epidemiology and Prevention of Vaccine-Preventable Diseases. U.S. Centers for Disease Control and Prevention. https://www.cdc.gov/pinkbook/hcp/table-of-contents/index.html. Accessed February 25, 2026.

Evans A, Chow S, Jennings R, et al. Traditional foods and practices of Spanish-speaking Latina mothers influence the home food environment: implications for future interventions. *J Am Diet Assoc*. 2011;111(7):1031-1038. doi:10.1016/j.jada.2011.04.007

Giralt A. Español médico y sociedad: Un libro para estudiantes de español en el tercer año de estudios. 2ª ed. Boca Raton, FL: Universal-Publishers; 2012. https://books.google.com/books?id=n1ewMQEACAAJ&printsec=frontcover&source=gbs_ge_summary_r&cad=0#v=onepage&q&f=false.

Juckett G. Caring for Latino patients. *Am Fam Physician*. 2013;87(1):48-54.

Longoria DA, Gonzalez JM, Escobar R, Rodriguez NM. Navigating care and self-care: Understanding Latina caregivers' prioritization and engagement in self-care practices. *J Transcult Nurs*. Published online February 14, 2026. doi:10.1177/10436596261423885

Martínez-Radl FB, Hinton DE, Stangier U. *Susto* as a cultural conceptualization of distress: Existing research and aspects to consider for future investigations. *Transcult*

REFERENCES/READING LIST

Mendenhall E, Fernandez A, Adler N, Jacobs EA. Susto, coraje, and abuse: Depression and beliefs about diabetes. *Cult Med Psychiatry*. 2012;36(3):480-492. doi:10.1007/s11013-012-9267-x

Moreira T, Hernandez DC, Scott CW, Murillo R, Vaughan EM, Johnston CA. Susto, coraje, y fatalismo: Cultural-bound beliefs and the treatment of diabetes among socioeconomically disadvantaged Hispanics. *Am J Lifestyle Med*. 2018;12(1):30-33. doi:10.1177/1559827617736506

Mossburg K, Moore-Beyioku C, Pico JR, Caruvana C, Acharya L. Evaluation of the use of herbs, spices, and supplements in the Hispanic/Latino/Latina population in Kokomo, Indiana, U.S.A. *J Community Health*. Published online December 21, 2025. doi:10.1007/s10900-025-01546-7

Obesity and Hispanic Americans. U.S. Department of Health & Human Services. https://www.minorityhealth.hhs.gov/omh/browse.aspx?lvl=4&lvlid=70. Updated January 2026. Accessed February 25, 2026.

REFERENCES/READING LIST

Ortiz CE, Smeltzer SC. Utilization of traditional healing practices from Mexico by U. S. women of Mexican origin: A systematic review. *Explore (NY)*. 2024;20(5):102997. doi:10.1016/j.explore.2024.04.001

Pachter LM, Weller SC, Baer RD, et al. Culture and dehydration: A comparative study of caída de la mollera (fallen fontanel) in three Latino populations. *J Immigr Minor Heal*. 2016;18(5):1066-1075. doi:10.1007/s10903-015-0259-0

Pinkey A, Anzuman M, Desai M, Eiring AM. Advances in the understanding of health disparities in the United States Hispanic population. *J Cancer Biol*. 2025;6(1):71-75. doi:10.46439/cancerbiology.6.076

¿Qué es un empacho? Farmacia Ecoceutics. https://www.ecoceutics.com/respuestas-de-salud/salud/empacho/. Accessed February 25, 2026.

Rush P, Houston P. *Spanish for Health Care*. 2nd ed. Upper Saddle River, NJ: Pearson Education; 2011.

Sanchez AA. An examination of the folk healing practice of curanderismo in the Hispanic community. *J Community Health Nurs*. 2018;35(3):148-161. doi:10.1080/07370016.2018.1475801

REFERENCES/READING LIST

Sarmiento I, Paredes-Solís S, Loutfi D, Dion A, Cockcroft A, Andersson N. Fuzzy cognitive mapping and soft models of indigenous knowledge on maternal health in Guerrero, Mexico. *BMC Med Res Methodol*. 2020;20(1):125. doi:10.1186/s12874-020-00998-w

Sass L, Alvarez E. Metaphor, magic, and mental disorder: Poetics and ontology in Mexican (Purépecha) *curanderismo*. *Transcult Psychiatry*. 2023;60(5):781-798. doi:10.1177/13634615211043769

Standards, Publications, and Notification Forms. Liaison Committee on Medical Education. https://lcme.org/publications/. Published 2024. Accessed February 25, 2026.

Stein MJ. Blood, sweat, and/or tears: Comparing nervios symptom descriptions in Honduras. *Cult Med Psychiatry*. 2019;43(2):256-276. doi:10.1007/s11013-018-9614-7

State of Health Among Hispanics in the US in 2022. Hispanic Information and Telecommunications Network. https://www.hitn.org/state-of-health-among-hispanics-in-the-us-in-2022/. Updated 2026. Accessed March 2, 2026.

REFERENCES/READING LIST

United States Census Bureau. Language Spoken at Home. https://www.census.gov/acs/www/about/why-we-ask-each-question/language/. Accessed February 25, 2026.

U.S. Department of Health and Human Services Office of Minority Health. Hispanic/Latino Health. https://minorityhealth.hhs.gov/hispaniclatino-health. Updated September 2025. Accessed February 25, 2026

Waldram JB, Hatala AR. Does "susto" really exist? Indigenous knowledge and fright disorders among Q'eqchi' Maya in Belize. *Cult Med Psychiatry*. 2023;47(2):372-401. doi:10.1007/s11013-022-09777-2

Waugh LJ. Beliefs associated with Mexican immigrant families' practice of la cuarentena during postpartum recovery. *J Obstet Gynecol Neonatal Nurs*. 2011;40(6):732-741. doi:10.1111/j.1552-6909.2011.01298.x

Weller SC, Baer RD, García de Alba García J, Salcedo Rocha AL. Susto and nervios: Expressions for stress and depression. *Cult Med Psychiatry*. 2008;32(3):406-420. doi:10.1007/s11013-008-9101-7

¿TIENEN PREGUNTAS?

Ask.librarian@wmed.edu
