# Supplementary material for: Developing a one-shot medical Spanish module for medical students: an open educational resource approach
Source: Front Med (Lausanne). 2026 Sep 17;13:1957506. doi: 10.3389/fmed.2026.1957506 (PMC13627033; doi:10.3389/fmed.2026.1957506)
Supplement: Supplementary file 2 [file Table1.docx]

| **Title:** Developing a One-Shot Medical Spanish Module for Medical Students: An Open Educational Resource Approach  **Journal:** Frontiers in Medicine  **Author:** Juli McCarroll  **Affiliation:** Western Michigan University Homer Stryker M.D. School of Medicine, Department of Medical Library, 1000 Oakland Drive, Kalamazoo, MI, United States of America  **E-mail:** [juli.mccarroll@wmed.edu](mailto:juli.mccarroll@wmed.edu) |  |  |  |
| --- | --- | --- | --- |
|  |  |  |  |
|  |  |  |  |
|  |  |  |  |
|  |  |  |  |
|  |  |  |  |
|  |  |  |  |
|  |  |  |  |

|  |  |  |  |
| --- | --- | --- | --- |
|  |  |  |  |
|  |  |  |  |

Ankle <https://commons.wikimedia.org/wiki/File:Ankle_highlighted.png>

Arms <https://upload.wikimedia.org/wikipedia/commons/f/ff/Proximal_rupture_repair.png>

Back <https://commons.wikimedia.org/wiki/File:Back.JPG>

Ear <https://commons.wikimedia.org/wiki/File:Closeup_of_a_human_ear.jpg>

Elbow <https://commons.wikimedia.org/wiki/File:Location_of_UCL_injury.jpg>

Eye <https://commons.wikimedia.org/wiki/File:Amber_eye_%F0%9F%91%81.jpg>

Feet <https://commons.wikimedia.org/wiki/File:Newborn-Baby-Feet.jpg>

Finger <https://commons.wikimedia.org/wiki/File:Index_finger_2.JPG>

Knees <https://commons.wikimedia.org/wiki/File:Left_knee_prepatellar_bursitis.jpg>

Legs <https://commons.wikimedia.org/wiki/File:1-legs-and-black-shoes.JPG>

Nails <https://commons.wikimedia.org/wiki/File:Fingernails.jpg>

Neck <https://commons.wikimedia.org/wiki/File:Sobo_1909_267.png>

Nose <https://commons.wikimedia.org/wiki/File:Nose_with_glasses.jpg>

Shoulder <https://commons.wikimedia.org/wiki/File:Shoulderjoint.PNG>

Stomach <https://commons.wikimedia.org/wiki/File:Stomach_pain.jpg>

Throat <https://commons.wikimedia.org/wiki/File:Pharyngitis,_patiens,_ICD-10_%D0%9234.1._16_years_old,_female.jpg>

**Dialogue/Diálogo**

A: Good morning, sir.	Good morning, ma’am.	Good morning, miss.

Buenos días, señor.	Buenos días, señora.	Buenos días, señorita.

A: My name is Dr. _____.

Soy el doctor _____/la doctora _____.	Me llamo Dr. _____/Dra. ____.

A: What’s your name?

¿Cómo se llama Usted (Ud.)?

B: I’m _____.

Soy _____.	Me llamo _____.

A: Nice to meet you. How are you feeling?

Mucho gusto. ¿Cómo se siente?

B: I don’t feel good.	I feel awful.

No me siento bien.	Me siento muy mal.

A: Are you in any pain?

¿Tiene algún dolor?

B: Yes, my ear hurts.	Yes, my head hurts.	Yes, my knee hurts.	Yes, my finger hurts.

Sí, me duele el oído.	Sí, me duele la cabeza.	Sí, me duele la rodilla.	Sí, me duele el dedo.

A: Let me take a look.

Déjeme verlo.	Déjeme verla.	Déjeme verla.	Déjeme verlo.

**Match the description to the condition**

1. **Causes**: stress, fright, family/spouse issues, getting angry

**Symptoms:** depression, worry, impatience, headache, toothache

**Treatment:** manzanilla tea, going to a traditional healer or pharmacy

2. **Causes**: eating too much or too fast/spoiled or greasy food/when you’re not hungry/on the run, eating and smoking, drinking alcohol/caffeine, swallowing gum

**Symptoms**: stomachache, bloating, cramps, diarrhea, vomiting, nausea, gas, dry mouth, tiredness, headache, perspiration

3. **Cause**: frightening event or experience that attacks the spirit and causes the soul to leave the body

**Symptoms:** insomnia, lethargy, lack of participation in or enjoyment of activities, detachment from life and people, nervousness, anorexia, fever, diarrhea

4. Latinos are 1.7 times more likely to have it and 1.4 times more likely to die from it
5. 40-day post-partum recuperation period to bond with baby and heal physically and emotionally
6. Used to treat colic, upset stomach, nausea, cramps, vomiting, sore throat
A. el empacho
B. la manzanilla
C. la cuarentena
D. la diabetes
E. el susto
F. los nervios

**Speaker Notes**

**Slide 2**

Good morning. Did everyone pick up a copy of the body parts handout, the dialogue, the matching exercise, and something to write with when you came in? You’ll need those in a few minutes.

Hola y bienvenidos a la clase de hoy donde daré un breve resumen del español y la cultura latina en el ámbito médico. In other words, hello and welcome to today’s class where I’ll give a brief overview of Spanish and the Latino culture in the medical setting.

The content I’ll cover today includes the terms *Hispanic* and *Latino* and it’s important to understand the difference. *Hispanic* focuses on language and culture and generally describes people who speak Spanish or trace their ancestry to a Spanish-speaking country, including Spain, whereas *Latino* focuses on geography and refers to individuals with roots in Latin America. Although these terms have different meanings, they are often used synonymously and not distinguished in the literature. Since the majority of Spanish-speaking patients you’ll encounter in the U.S. are Latino, I’ll use that term. There are also many dialects of Spanish, and I’ll use a standard conservative version, as opposed to Castilian Spanish from Madrid or radical dialects from the Caribbean, Andalusia, and Chile.

**Slide 3**

I have no conflicts of interest.

**Slide 4**

This medical Spanish module is licensed under a Creative Commons Attribution–NonCommercial 4.0 International License (CC BY-NC 4.0), which allows users to distribute, remix, adapt, and build upon the material in any medium or format, for noncommercial purposes. You must give appropriate credit, provide a link to the license, and indicate if changes were made. You can view the license at <https://creativecommons.org/licenses/by-nc/4.0/>.

**Slide 5**

After today’s event, you will be able to: 1. Describe four conditions that Latino patients may present with, 2. In Spanish, introduce yourself to a Spanish-speaking patient and ask how they’re feeling, and 3. Name two Spanish translation resources you can use for further learning.

**Slide 6**

Here’s a little bit about me. I have been using Spanish to communicate since I took my first Spanish class in high school. I went on to complete my senior year of high school in Spain. Here I am with my Spanish host family, backpacking through the north of Spain on the Camino de Santiago pilgrimage trail, at the Chichén Itzá Mayan ruins in Mexico, the Sagrada Familia church in Barcelona, and the Christopher Columbus Plaza in San Juan Puerto Rico. I later got Bachelor’s and Master’s degrees in Spanish, Linguistics, and Library and Information Science and have used Spanish in a variety of professional settings, including managing an emergency shelter for Latino families, teaching for Migrant Head Start and other organizations, running a Medicaid outreach program in the Latino community, and serving as an elevator mechanic dispatcher to release Spanish-speaking passengers stuck in elevators.

How about you? What’s your experience using Spanish? Raise your hand if you understand no Spanish. Now raise your hand if you understand a little bit. And finally, raise your hand if you’re bilingual in Spanish. You can see we have a wide variety of Spanish language abilities represented here, and we’ll do some work in the next hour to understand and communicate more effectively with Spanish speakers.

**Slide 7**

There are many reasons to learn medical Spanish. In general, it helps promote health equity and reduce health disparities. Specifically, LCME Standard 7.6 is related to culturally competent healthcare, healthcare disparities and inequities, and skills needed to provide care to diverse populations. Standard 7.8 is related to the skills needed to communicate with patients, families, and colleagues.

Next, according to the US Census Bureau, as of 2026, 14% of the US population speaks Spanish, making it the second-most-commonly-spoken language after English, so it’s likely that you’ll treat patients who speak only Spanish or speak English with varying degrees of fluency. Patient-physician language concordance has been shown to improve communication, patient safety and satisfaction, appointment and prescription adherence, and health outcomes.

Also, it is important to know your linguistic and cultural limitations and only use Spanish in the medical setting appropriately, calling on qualified interpreters when needed, since limited proficiency can lead to miscommunication and errors that can compromise patient safety.

Learning Spanish allows you to increase your cultural knowledge, understanding, and respect. Cultural humility is the ability to develop the knowledge, skills, and attitude needed to deliver health care services that meet the social, cultural, and linguistic needs of patients, regardless of their backgrounds. Cultural competence doesn’t have an endpoint, but is more of a process or a goal. It’s a good idea to be familiar with service area population demographic information so you can brush up on your cultural competency skills, maybe by getting involved with community medical projects or agencies/programs that serve different demographic groups.

Instead of focusing on memorizing vocabulary, practice practical communication skills like introductions, obtaining consent, assessing pain, answering medication questions, taking a history, answering patient questions, educating patients, and confirming patient understanding.

I encourage you to continue learning medical Spanish, look for formal proficiency assessments, use interpreters appropriately, take Spanish electives, and get involved with Spanish speakers in the community.

**Slide 8**

We’re going to switch gears and look at body parts vocabulary that will be useful to know when speaking with your patients. Is anyone familiar with the game lotería? It sounds similar to the English word lottery, but with Spanish pronunciation-lotería. It’s similar to a Mexican version of BINGO. Traditionally the board has images representing Mexican folk art, culture, and daily life, but today, we’re going to play a modified version that has body parts on it. I’ll say a body part in Spanish, and then you repeat it, I’ll say it again, and you repeat it again. Then, look at your lotería board and it you have the picture of that body part, write the name of the body part IN SPANISH on the image. When you have 4 pictures marked in a row, horizontally, diagonally, or vertically, yell lotería! I’ll check to make sure you have written in Spanish 4 of the parts called in a row, and if so, you are the winner. Look at the board and make sure you know which body part each picture is trying to represent. We have nails, knees, elbow, foot, leg, arm, finger, neck, stomach, ear, eye, throat, nose, shoulder, ankle, and back. Do you have any questions?

And the first one is…

**Slide 9**

Las rodillas. Now it’s your turn. (Point toward students and they repeat). Las rodillas. (Point toward students and they repeat).

Spanish is a gendered language and most feminine nouns end in a or ion and masculine nouns end in o, ma, l, r, ne, or e. Plurals end in s or es.

**Slide 10**

Next is el nariz. (Point toward students and they repeat). El nariz. (Point toward students and they repeat).

**Slide 11**

El oído. (Students repeat). El oído. (Students repeat).

**Slide 12**

El ojo. (Students repeat). El ojo. (Students repeat).

**Slide 13**

La garganta. (Students repeat). La garganta. (Students repeat).

**Slide 14**

Las uñas. (Students repeat). Las uñas. (Students repeat).

**Slide 15**

El hombro. (Students repeat). El hombro. (Students repeat).

**Slide 16**

El tobillo. (Students repeat). El tobillo. (Students repeat).

**Slide 17**

El codo. (Students repeat). El codo. (Students repeat).

**Slide 18**

Los pies. (Students repeat). Los pies. (Students repeat).

**Slide 19**

Las piernas. (Students repeat). Las piernas. (Students repeat).

**Slide 20**

Los brazos. (Students repeat). Los brazos. (Students repeat).

**Slide 21**

El dedo. (Students repeat). El dedo. (Students repeat).

**Slide 22**

El cuello. (Students repeat). El cuello. (Students repeat).

**Slide 23**

El estómago. (Students repeat). El estómago. (Students repeat).

**Slide 24**

La espalda. (Students repeat). La espalda. (Students repeat).

(When someone yells lotería, have them say the words they marked on their board in Spanish and make sure those words were called. If not, continue the game.)

Congrats! ¡Felicidades! Here’s your prize-some popular candy from Mexico.

(Place the rest of the candy at the exit for students to take as they leave).

**Slide 25**

We've talked about parts of the body. Now we’ll talk about some typical conditions or dolencias that Latino patients may present with or practices they may or not follow. Just like within or between any groups of people, not all Spanish speakers are the same, and it’s important not to stereotype or treat the group as a whole, but to treat all patients as individuals.

The top causes of death among Latinos are cancer and heart disease and the most common conditions are obesity, hypertension, and diabetes.

We’ll also look at some conditions seen more commonly in the Latino community including nerves, indigestion, fright, and the practice of quarantine, and talk about common home remedies and attitudes about vaccinations.

**Slide 26**

We’ll also look at some conditions seen more commonly in the Latino community including nerves, indigestion, fright, and the practice of quarantine, and talk about common home remedies and attitudes about vaccinations.

Los nervios is a folk illness caused by cultural influences and is not a virus or physical illness. It can present like PTSD, depression, stress, schizophrenia. It can develop in children as a result of trauma or in adults due to stress or hardship. Causes include stress, fright, family issues, or being angry. Symptoms include depression, worry, impatience, headache, and toothache. Treatments include chamomile tea or going to a traditional healer or pharmacy. Many people are not comfortable seeking out mental health services because of what others might think. They might use the term nervios instead of depression, anxiety, etc. If your patient uses the term nervios, ask questions to find out exactly what they’re referring to.

**Slide 27**

El empacho is a culturally-bound folk illness with many possible causes, symptoms, and cures. It presents like severe indigestion, dyspepsia, or gastroparesis. Causes include eating too much or too fast/spoiled or greasy food/when you’re not hungry/on the run, eating and smoking, drinking alcohol/caffeine, and swallowing gum. Symptoms include stomachache, bloating, cramps, diarrhea, vomiting, nausea, gas, dry mouth, tiredness, headache, and perspiration. Prevention includes eating less food more often, starting the meal with soup or salad, drinking more water, not going to bed right after eating, chewing slowly, avoiding fried food and heavy sauces, stopping eating before you’re full, and exercising regularly.

**Slide 28**

El susto is a culturally-bound illness that can present like depression, PTSD, or a panic attack. Like nervios, can be used to describe schizophrenia. The cause is usually a frightening event or experience that attacks the spirit and causes the soul to leave the body. Symptoms include insomnia, lethargy, lack of participation in or enjoyment of activities, detachment from life and people, nervousness, anorexia, fever, and diarrhea. Treatment includes ceremonies with a traditional healer. It’s important to talk to the patient about the event that caused the onset.

**Slide 29**

La cuarentena is a 40-day post-partum recuperation period for mom to bond with baby and heal physically and emotionally. It may sound like the English quarantine and remind you of COVID-19 quarantine, but this is a different kind of quarantine. During this time, mothers are given support by female family and community members so they can rest and recuperate. This time breastfeeding more convenient, especially right after birth so baby can build immunity with colostrum. Mothers may be superstitious about this period and not want to violate its principles, so you may notice they are not making medical appointments or making them and not coming in. Ask mom if she is adhering to la cuarentena to determine if she’s staying home for that or if she may be experiencing post-partum depression.

**Slide 30**

It’s important to remember that Latinos, like many people, may use home remedies in addition to medicine from traditional healers or curanderas, pharmacies that stock traditional treatments and modern prescription drugs, as well as having strong religious beliefs. Some believe in fatalism, or that fate is pre-destined, so they may not believe in treatment or drugs being able to make them better.

AARP conducted a study on Latinos’ use of home remedies. It found that about two-thirds of Latinos 45 and older use home remedies based on foods and herbs. Patients may not view these as medicinal, so if you ask them what medication they’re taking, they may not mention them. Ask directly if they’re using any home remedies so you have a clear picture and don’t prescribe anything that could cause drug interactions. The study also found that 65% of Latinos age 45 and older believe herbal remedies and supplements are effective and 62% use them occasionally, 27% at least once/week, and 16% daily. Use is higher among those not born in the U.S. 70% did not consult a doctor before starting home remedies and some use home remedies instead of prescribed medication. Other studies look at medicinal plant use for inflammatory and neuropathic pain and remedies to prevent and mitigate COVID-19 symptoms.

**Slide 31**

Here are some home remedies that Latinos may use for different conditions. Chamomile/la manzanilla is used for colic, upset stomach, nausea, cramps, and vomiting, while aloe/la sábila is for burns, sores, acne, and cuts. Rue/la ruda is effective for earaches and anise/el anis for fright or el susto. Mint/la hierbabuena is a typical herb for colds, colic, upset stomach, nerves or los nervios, and intestinal issues, while wormwood/el ajenjo is helpful for gas, colic, diarrhea, and nausea. Orange tree leaf tea/el té de Naranjo can help with cramps, nerves, and insomnia and basil/la albahaca for fright or el susto. Oregano/el oregano can be used for the flu, colds, sore throat, or congestion and garlic/el ajo is helpful for stomachache, insomnia, insect bites, arthritis, and earaches. Finally, Vick’s VapoRub is used for shortness of breath, laryngitis, sinuses, colds, flu, and many other ailments.

**Slide 32**

Overall, Latinos have lower vaccine rates than the overall population, and rates vary depending on location, employment status, and income.

Reasons that vaccine uptake may be lower include misinformation, patient-physician language discordance, limited health literacy in English, limited access to primary care, high cost of care, lack of health insurance, inflexible work schedules, immigration fears, transportation issues, lack of Latino-specific outreach, cultural beliefs and perceptions of vaccine necessity, lower trust in the healthcare system due to prior negative experiences. Also, migrant families who move frequently may have a hard time keeping vaccination records from all of the places they’ve lived, if they received them.

Some people believe illnesses have been eradicated and vaccines are no longer needed, vaccines cause disease, their kids don’t need to get vaccinated as long as the other kids around them are, or vaccines are not needed until kids are older. However, you can see from this data from the Department of Health and Human Services that vaccines have had a large positive impact on morbidity.

It can be helpful to emphasize the communal impact of vaccines since Latinos tend to be community and collectivist oriented. Being part of a group and having extended family support is common and this may be evident when people not genetically related to the patient accompany them on visits.

**Slide 33**

Now let’s see what you remember. Take out the matching exercise. You have 1 minute to match the description to the term.

**Slide 34**

Who can answer number 1? Number 2? Number 3? Number 4? Number 5? Number 6?

**Slide 35**

Very good. Once again, here are the answers. Do you have any questions?

**Slide 36**

You’ve done a lot of listening, so let’s get talking! We have a brief dialogue between a patient and doctor. Señorita is generally used for younger, unmarried women, while señora is for older, married women, and señor for men. You’ll introduce yourself to the patient and find out what brought them into the office. Part A is the doctor and Part B is the patient. You’ll notice there are blanks in some of the phrases. You can fill those in with your name. I’ll read each phrase twice and then you repeat. Then I’ll play the doctor and pair up with one of you to demonstrate to the group. Then I’ll play the patient and pair up with one of you to demonstrate to the group. Then you’ll pair up and do the dialogue with a partner. For this dialogue example, I’ll be the doctor and use Dr. Smith since that’s easier than my last name, and my patient is John. We’ll do half of the dialogue at a time, so we’ll do just this slide for now.

I’ll start and say the first phrase twice and you can repeat after me. Buenos días, señor. Buenos días, señor. (Students repeat as a group.) Buenos días, señora. Buenos días, señora. (Students repeat as a group.) Buenos días, señorita. Buenos días, señorita. (Students repeat as a group.) Soy la doctora Smith. Soy la doctora Smith. (Students repeat as a group.) ¿Cómo se llama Usted? ¿Cómo se llama Usted? (Students repeat as a group.) Me llamo John. Me llamo John. (Students repeat as a group.) Mucho gusto. Mucho gusto. (Students repeat as a group.) ¿Cómo se siente? ¿Cómo se siente? (Students repeat as a group.)

You’re doing great with the pronunciation. Now, who would like to model the pronunciation with me? (Choose a student and have them repeat each line of the dialogue after you.)

Great job listening and pronouncing the phrases. Now I’ll be the doctor. Who wants to be the patient with me? (Model dialogue for the group).

Now I’ll be the patient. Who wants to be the doctor with me? (Model dialogue for the group).

Now pair up with a partner to do the dialogue. One of you is the doctor and the other is the patient. Feel free to use any of the options in the phrases that have them. Go through it once and then we’ll stop and see if there are any questions before switching roles. Remember, you don’t have to pronounce things perfectly. Just do your best and help each other remember how pronounce the words. Try to look at your paper less each time you go through it. Any questions? OK, go through the dialogue once.

Great! Do you have any questions? Now switch roles and go through the dialogue again.

**Slide 37**

Now we’ll move on to the second part of the conversation. Once again, repeat after me. No me siento bien. No me siento bien. (Students repeat as a group.) Me siento muy mal. Me siento muy mal. (Students repeat as a group.) ¿Tiene algún dolor? ¿Tiene algún dolor? (Students repeat as a group.) Sí, me duele el oído. Sí, me duele el oído. (Students repeat as a group.) Sí, me duele la cabeza. Sí, me duele la cabeza. (Students repeat as a group.) Sí, me duele la rodilla. Sí, me duele la rodilla. (Students repeat as a group.) . Sí, me duele el dedo. . Sí, me duele el dedo. (Students repeat as a group.)

You’re doing great with the pronunciation. Now, who would like to model the pronunciation with me? (Choose a student and have them repeat each line of the dialogue after you.)

Great job listening and pronouncing the phrases. Now I’ll be the doctor. Who wants to be the patient with me? (Model dialogue for the group).

Now I’ll be the patient. Who wants to be the doctor with me? (Model dialogue for the group).

Now pair up with a partner to do the dialogue. One of you is the doctor and the other is the patient. Feel free to use any of the options in the phrases that have them. Go through it once and then we’ll stop and see if there are any questions before switching roles. Remember, you don’t have to pronounce things perfectly. Just do your best and help each other remember how pronounce the words. Try to look at your paper less each time you go through it. Any questions? OK, go through the dialogue once.

Great! Do you have any questions? Now switch roles and go through the dialogue again.

That sounded good. (If there is enough time). Let’s switch again and this time use different options than you did the first time and try not to look at your paper. And switch one final time and use different options than you did last time and try not to look at your paper.

Now I need 2 people to demonstrate for the group. (Choose a pair to demonstrate.)

Is there anyone else who wants to model the dialogue for the group? (Choose another pair if there is time and interest.)

Now you can introduce yourself and ask how your patient is feeling.

**Slide 38**

Let’s take a break from speaking and look at some translation resources that will be helpful for continued learning once you leave today. These are three common translation resources with links for the websites. CanopySpeak is the only one that is specifically geared to the medical setting. The translation capabilities are all web-based and app-based, except for CanopySpeak, which only has an app. You can look up words or phrases, listen to the pronunciation, and repeat it.

(Open hyperlinks on the computer for Google Translate and SpanishDict websites. Look up “How are you feeling?” and click the audio button to play. Then open the Google Translate and SpanishDict apps on your phone, search for “How are you feeling”, and play the phrases. For CanopySpeak, click on the computer hyperlink to show what the website looks like and play the phrase from the app on your phone.)

**Slide 39**

You have received a lot of information in the past hour. Here are some steps you can take if you are interested in Spanish or your future career is in a setting that has Spanish-speaking patients. And of course this applies not only to Spanish, but any other language or population you are interested in learning more about or serving.

First, check out the references and reading list for more useful information about treating Spanish-speaking patients. Then download and practice your speaking, reading, and listening with the Canopy Speak app. Canopy was made for the medical setting so it uses the most appropriate language for that setting. It’s an easy way to practice Spanish when you’re on the go or during away rotations. We’ve had about 250 students go through the Canopy medical Spanish elective here at the school and have received feedback that the app is very useful. You can also get involved in the community with any groups or agencies that provide service to the Spanish-speaking community to increase your cultural competency skills. When doing clerkships, if you're interested in working with a Spanish-speaking population, ask Educational Affairs if they can identify a site for you and place you there. You can also enroll in an M4 Spanish elective offered by the library. There is Advanced Canopy levels 1, 2, and 3 and WikiProject Medical Translation. They are online and designed to be flexible and fit your schedule. These can be helpful with residency applications because they demonstrate your commitment to providing linguistically and culturally concordant care. Have fun with the language through music, movies, comics, food, games, books, media, or culture. Whatever you like to do, do it in Spanish. It will benefit you and your future patients.

**Slide 40**

Let’s look back at our learning objectives for today. Can someone describe four conditions that Latino patients may present with? Is there someone who would like to play the role of a doctor and introduce yourself in Spanish to a Spanish-speaking patient and ask how they’re feeling? And can somebody name two Spanish translation resources you can use for further learning? (Learning and recall will also be assessed with two embedded questions on the upcoming summative exam covering information in this learning event.)

Thank you for actively participating in this brief medical Spanish session. The PowerPoint is available to you in the learning management system. If you’re interested in getting more involved with medical Spanish, the resources in the reading list are useful. (Scroll through the reading list in slides 41-47.)

**Slide 48**

Last call for questions! If you think of something later about medical Spanish or library Spanish electives, please contact me. And feel free to grab some candy on your way out.
